# From Corn Starch to Nanostructured Magnetic Laser‐Induced Graphene Nanocomposite

**DOI:** 10.1002/smll.202405252

**Published:** 2024-10-18

**Authors:** Sreenadh Thaikkattu Sankaran, Alexander Dallinger, Anna Chiara Bressi, Attilio Marino, Gianni Ciofani, Aleksandra Szkudlarek, Vitaliy Bilovol, Krystian Sokolowski, Birgit Kunert, Hana Kristin Hampel, Hilda Gomez Bernal, Francesco Greco

**Affiliations:** ^1^ The Biorobotics Institute Scuola Superiore Sant'Anna Viale Rinaldo Piaggio 34 Pontedera 56025 Italy; ^2^ Department of Excellence in Robotics & AI Scuola Superiore Sant'Anna Piazza Martiri della Libertà 33 Pisa 56127 Italy; ^3^ Institute of Solid State Physics NAWI Graz Graz University of Technology Petersgasse 16 Graz 8010 Austria; ^4^ Smart Bio‐Interfaces, Istituto Italiano di Tecnologia Viale Rinaldo Piaggio 34 Pontedera 56025 Italy; ^5^ Academic Centre for Materials and Nanotechnology AGH University of Krakow av. Mickiewicza 30 Krakow 30‐059 Poland; ^6^ Institute of Experimental Physics NAWI Graz Graz University of Technology Petersgasse 16 Graz 8010 Austria; ^7^ Interdisciplinary Center on Sustainability and Climate Scuola Superiore Sant'Anna Piazza Martiri della Libertà 33 Pisa 56127 Italy

**Keywords:** biodegradables, bioderived, core–shell nanoparticles, iron‐catalytic, laser‐induced graphene, magnetic, starch

## Abstract

Laser‐Induced Graphene (LIG) is a 3D, conductive, porous material with a high surface area, produced by laser irradiation of synthetic polymers with high thermal stability. Recently, the focus has shifted toward sustainable bioderived and biodegradable precursors, such as lignocellulosic materials. Despite starch being an abundant and cost‐effective biopolymer, direct laser scribing on starch‐derived precursors has not yet been explored. This study demonstrates that corn starch bioplastic (SP) can be converted into LIG through iron‐catalyzed laser‐induced pyrolysis, using Fe(NO₃)₃ as an additive. The impact of iron additive concentration on LIG formation and on its properties is investigated, with only certain concentrations yielding reliable and reproducible results. The investigation of LIG's crystal structure reveals magnetic and non‐magnetic iron phases: γ‐Fe₂O₃, Fe₃C, and Fe(C). The LIG nanocomposite exhibits soft magnetic properties, with a coercive field of H_c_ ≈ 200 Oe and a saturation magnetization of M_s_ ≈ 67 emu g⁻¹. The SP substrate degrades almost entirely in soil within 12 days and is unaffected by the addition of Fe(NO₃)₃, allowing for material compostability in line with circular economy principles. Consequently, SP stands out as a promising “green” precursor for magnetic LIG, paving the way for sustainable applications in environmental remediation.

## Graphical abstract

Corn starch bioplastic (SP) can be converted into laser‐induced graphene (LIG) via iron‐catalyzed pyrolysis, using Fe(NO₃)₃. Varying the Fe(NO₃)₃ concentration affects LIG properties. The obtained nanocomposite of LIG with core–shell Fe_3_C─C particles shows soft magnetic properties. SP almost completely degrades in soil within 12 days. This method enables bioderived magnetic LIG production for environmental applications.

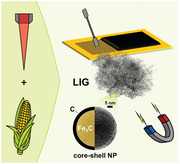

## 1 Introduction

The widespread availability of low‐cost laser devices has led to a significant increase in the use of laser‐based processing and manufacturing techniques. Direct laser writing (DLW) can be sorted into three categories: subtractive, additive, and transformative DLW. In subtractive DLW, the material is selectively removed from the substrate through techniques like ablation or etching, such as micromachining. In contrast, additive DLW consists of creating complex structures by adding material onto a substrate, often achieved via photon‐induced polymerization, as in the case of 3D printing. Instead, transformative DLW is a peculiar case where laser irradiation induces changes in the chemical or structural properties of the precursor material. This process occurs without significant ablation or the need for additional chemicals or a specialized environment.^[^ ^1^ ^]^

One material created by transformative DLW is Laser‐Induced Graphene (LIG), produced by laser‐induced pyrolysis when a polymer substrate is irradiated with a laser. This method has been known since the late 1980s,^[^ ^2^ ^]^ but was reinvestigated in detail in 2014^[^ ^3^ ^]^ and its graphene‐like structure was discovered. This technique consists of a rapid photothermal (and photochemical) process carried out with commercially available laser engravers. The properties of LIG can be tuned by changing the laser parameters, environmental conditions, and the precursor material and its composition. It is possible to change the electric conductivity, porosity, surface properties, morphology (from porous to fiber‐like structures), chemical composition, and others.^[^ ^4^, ^5^ ^]^

The easy and fast production process and tunable properties make LIG a suitable candidate for applications in various fields. LIG has been used for physical sensors (piezoresistive sensors for strain and pressure, chemiresistive gas sensors, photoelectric, temperature and humidity sensors),^[^ ^6^ ^]^ chemical sensors (without and with functionalization for detecting uric acid, tyrosine, dopamine, glucose, salmonella, and others),^[^ ^7^ ^]^ energy devices such as lithium‐ion batteries,^[^ ^8^ ^]^ supercapacitors^[^ ^9^, ^10^ ^]^ and triboelectric nanogenerators,^[^ ^11^ ^]^ and actuators for soft robotics.^[^ ^12^ ^]^

Moreover, it was recently discovered that nanocomposites from LIG can develop soft magnetic properties (*H_c_* = 74–310 Oe), achieved by either mixing the precursor material with Iron(III) acetylacetonate (Fe(acac)_3_),^[^ ^13^, ^14^ ^]^ drop casting it on LIG with subsequent reirradiation^[^ ^15^ ^]^ or electroplating with Ni nanoparticles.^[^ ^16^ ^]^ These magnetic LIG nanocomposites were used for electromagnetic interference shielding^[^ ^13^, ^15^, ^16^ ^]^ and dye removal.^[^ ^14^ ^]^

A significant advantage of LIG nanocomposites in comparison to more conventional methods for the preparation of magnetic carbon nanomaterials is the advancement in terms of process simplicity and the use of fewer problematic chemicals. Nowadays, conventional magnetic carbon nanomaterials can be synthesized via a variety of methods, including hydrothermal, ultrasonic‐sonochemical, and microwave methods.^[^ ^17^ ^]^ Their applications include catalysis, environment remediation, energy storage, medical sciences, and removing dyes and heavy metals from wastewater^[^ ^18^ ^]^ and have the advantage that they can easily be magnetically separated and recovered without the need for centrifugation or extensive filtration.^[^ ^17^, ^19^, ^20^, ^21^ ^]^ However, despite being more complex and using more chemicals, conventional methodologies have effectively illustrated the viability of employing sustainable materials, such as agricultural byproducts, including peels and shells^[^ ^22^ ^]^ or sugarcane bagasse,^[^ ^23^ ^]^ as precursors for activated carbon or graphene oxide.^[^ ^24^ ^]^

Implementing a one‐step process, such as laser‐induced pyrolysis, to create magnetic LIG nanomaterials from bioderived precursors would represent a significant advancement in synthesizing such materials.

In recent years the research focus on LIG has included bioderived and biodegradable precursors.^[^ ^9^, ^25^ ^]^ The vast majority of “green” precursors for LIG consist of lignocellulosic materials (i.e., containing lignin, hemicellulose, and cellulose), which are widely abundant on Earth since they are the major constituents of plant cell walls.^[^ ^26^ ^]^ Various approaches for different LIG precursors such as wood, cork, and paper, as well as refined biopolymers (lignin and nanocellulose) extracted from bioderived materials, were investigated. Most of them required different protocols for effective carbonization, such as multiple lasering, defocusing, scribing in inert atmospheres, and/or specific pretreatments.^[^ ^9^, ^25^ ^]^

When evaluating bioderived precursors, starch‐based materials should be considered, since they can be produced cost‐effectively in large quantities and are readily available from common crops such as potatoes and corn. In addition to these characteristics, the use of starch as a precursor offers the additional benefits of its solubility in water, its capacity to form cohesive films, and its good biodegradability and biocompatibility.^[^ ^27^ ^]^ These characteristics make it an excellent replacement for synthetic polymers in a variety of applications in health and medicine,^[^ ^28^, ^29^ ^]^ engineering,^[^ ^28^ ^]^ alternative packaging,^[^ ^30^ ^]^ and as a suitable substrate for green electronics.^[^ ^28^, ^31^ ^]^ In the latter, several studies focused on tailoring starch‐based substrates (e.g., bioplastic films, starch‐based gels) to be soft, stretchable, foldable, transparent, and compliant with flexible electronics requirements.^[^ ^3^, ^28^, ^31^, ^32^ ^]^ Furthermore, starch has also been investigated to create magnetic nanocomposites for drug removal from aqueous solutions,^[^ ^33^ ^]^ water remediation,^[^ ^34^ ^]^ and drug delivery.^[^ ^35^ ^]^

To the best of our knowledge, a direct synthesis of starch into LIG has not been investigated yet.

The initial objective of this research was to develop a biodegradable LIG on a starch‐based bioplastic for stretchable electronics, which had not been demonstrated before. However, the electrical and mechanical properties of the starch‐based biopolymer proved to be inadequate to achieve this goal. Nonetheless, the objective of producing bioderived LIG from starch was accomplished, and the resulting material exhibited several unexpected and intriguing properties.

Given that laser writing on starch, and bioplastic resulted in just ablation, the addition of various metal nitrates to the bioplastic formulation was investigated. This was prompted by some evidence that especially iron nitrate seemed to decrease the disorder of LIG from wood, although this finding was not discussed by the authors.^[^ ^36^ ^]^ In combination with the knowledge that iron‐catalyzed graphitization of biomass with iron nitrate (Fe(NO_3_)_3_) was successfully demonstrated,^[^ ^37^ ^]^ this idea seemed a promising approach for laser‐induced pyrolysis.

The tested metal salts indeed facilitated the formation of LIG, but the primary investigation focused only on iron nitrate (Fe(NO_3_)_3_) since it was found to be the most effective. The impact on the LIG synthesis of Fe(NO_3_)_3_ and its concentration in the corn starch bioplastic (SP) was analyzed using techniques like thermogravimetric analysis (TGA), scanning electron microscopy (SEM), energy dispersive X‐ray analysis (EDS/EDX), and Raman spectroscopy. The degradability in the soil of these materials is assessed by mass loss and Fourier transform infrared spectroscopy (FTIR).

Further investigations on the starch‐derived LIG revealed the presence of iron compounds, whose nature and composition have been assessed through X‐ray diffraction analysis (XRD), transmission electron microscopy (TEM), high‐resolution TEM (HR TEM), selected area electron diffraction (SAED) and ^57^Fe Mössbauer spectroscopy. These in‐depth studies highlighted the presence of magnetic nanophases, which were then analyzed with vibrating‐sample magnetometry (VSM).

In this study, we present the first example of bioderived magnetic LIG nanocomposites, which could have applications in catalysis, environment remediation, energy storage, or medical sciences.^[^ ^18^ ^]^ The LIG precursor substrate can be composted, potentially reducing its environmental impact compared to synthetic precursors.^[^ ^38^ ^]^

## 2 Results and Discussion

### 2.1 LIG formation

**Figure 1:**
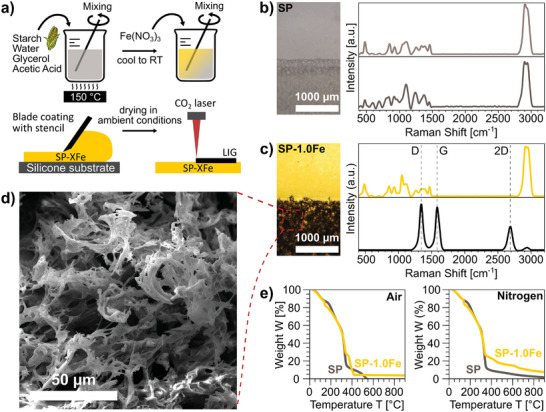
a) Schematic of preparation of SP and SP‐XFe: mixing and heating the ingredients, adding the iron nitrate, blade coating onto a silicone sheet with a stencil, and laser scribing; b) optical image of SP scribed with *P* = 15% and defocus *z* = 0 mm, and corresponding Raman spectra of the pristine (top) and scribed (bottom) area; c) optical image of SP‐1.0Fe scribed with *P* = 15% and *z* = 0 mm, and corresponding Raman spectra of the pristine (top) and scribed (bottom) area; d) SEM image of SP‐1.0Fe‐derived LIG; e) TGA curves for SP and SP‐1.0Fe in air and nitrogen environment.

The fabrication procedure detailed in this study involved the preparation of SP, as outlined in Methods (**Figure** **1**a). The SP is made of starch, deionized water, glycerol, and acetic acid. The presence of water helps the swelling and gelatinization of starch granules, and it also acts as a plasticizing agent along with glycerol, which plays a pivotal role in maintaining film flexibility. Acetic acid releases acetate and hydrogen ions in the starch mixture, which promote the disruption of amylopectin molecules,^[^ ^39^ ^]^ leading to lower gelatinization temperature as well as lower viscosity of gels,^[^ ^40^ ^]^ thus resulting in a more uniform cast film.

The pale grey and translucent SP film was laser‐scribed with different laser parameters (power, speed, and focus), as reported in Methods. The process only results in ablation and does not lead to any visible LIG formation. Indeed, this visual observation is confirmed by the Raman spectra of SP before and after ablation, which display the same characteristic bands, without any signs of carbonization (Figure 1b).

The addition of 1.0 mmol g^−1^ of Fe(NO_3_)_3_ resulted in the formation of a softer, yellow, and translucent film (SP‐1.0Fe). Its Raman spectrum displays a sharp peak at ≈1000 cm^−1^, which is characteristic of Fe(NO_3_)_3_.^[^ ^41^ ^]^ This peak is observed alongside the characteristic features of the pristine SP. SP‐1.0Fe was then scribed with the aforementioned laser settings and successfully transformed into LIG (Figure 1c). This finding demonstrates that iron‐catalyzed graphitization of starch is feasible via laser‐induced pyrolysis, which is comprehensively addressed in the section “Mechanism of iron‐catalyzed laser‐induced pyrolysis”.

It is also important to notice that the residual water content in SP‐XFe samples can impact pyrolysis, as its evaporation consumes a significant amount of energy that would otherwise be available for graphitization.

An estimation of LIG thickness (200 ± 40 µm) via a cross‐section of a scribed sample is provided in Supporting Information (Figure S1, Supporting Information).

The Raman spectrum shows the distinct bands of graphenic/graphitic materials,^[^ ^3^, ^42^ ^]^ with peaks D, G, and 2D found at 1344, 1584, and 2686 cm^−1^, respectively (Figure 1c).

The intensity ratios for SP‐1.0Fe with *I*_D_/*I*_G_ = 1.1 and *I*_2D_/*I*_G_ = 0.6 are slightly different from the ones of the benchmark PI‐derived LIG, which were 1.2 and 0.7, respectively.^[^ ^3^ ^]^ The *I*_D_/*I*_G_ ratio is proportional to the defects in the hexagonal structure of graphene, and thus a lower value is associated with a less‐defective structure.^[^ ^43^ ^]^ The *I*_D_/*I*_G_ ratio can be used to evaluate the crystalline size, according to Equation 1 (Experimental Section) and it is found to be *L_a_* = 17 nm. Instead, *I*_2D_/*I*_G_ is inversely related to the number of stacked layers, with monolayer graphene having *I*_2D_/*I*_G_ ≥ 2.^[^ ^45^ ^]^ The microscopic structure of the SP‐1.0Fe‐derived LIG consists of sparse entangled fibers emerging from the film (Figure 1d). In contrast, PI‐derived LIG consists of randomly oriented graphene sheets in a pore‐like structure.^[^ ^3^ ^]^

TGA was carried out to assess the material's thermal decomposition to understand the different behavior observed between SP and SP‐1.0Fe (Figure 1e) upon laser scribing. Since thermal damage and ablation rates are decreased under oxygen‐free atmospheres,^[^ ^46^ ^]^ the study has been conducted in both air and nitrogen environments. While SP was fully degraded at temperatures exceeding 400 °C, with no residual solid remaining in either air or nitrogen, SP‐1.0Fe exhibited a residual weight in both air and nitrogen environments. The addition of Fe(NO₃)₃ in SP‐1.0Fe resulted in a mass loss at 125 °C for both air and nitrogen atmospheres, as well as at 370 °C for an air atmosphere, which was not observed previously. Instead, the weight losses at 195 and 325 °C, already present in SP, were reduced for SP‐1.0Fe, and the final mass loss at 520 °C observed for SP was no longer evident. It should be considered that the heating associated with laser scribing is characterized by a much higher maximum temperature and by a shorter time scale with respect to what can be emulated with a TGA experiment, and this may affect thermodynamics. In practice, replicating the true thermal and environmental conditions of laser pyrolysis within a TGA experiment is impossible: the temperature reached during the scribing (2500 − 3000) K, the extremely fast rate of heating, and the local pressure and gas composition are arduous to be estimated,^[^ ^47^, ^48^ ^]^ let alone to be reproduced in a lab setup to provide reliable quantitative estimations of thermal degradation. Nevertheless, the data obtained from TGA with a scan rate of 10 °C min^−1^ within the specified temperature range provides at least a qualitative indication in support of the hypothesis that the addition of Fe(NO_3_)_3_ results in enhanced thermal stability of SP‐1.0Fe, which is a contributing factor for enabling the laser‐induced pyrolysis.

In addition to Fe(NO_3_)_3_, other nitrates with metals in the fourth row of the periodic table (Cr, Ni, Co, Cu), as well as iron (III) chloride, were also investigated to analyze their role in LIG synthesis (Figure S2a,b, Supporting Information). All the tested metal salts enable the laser‐induced pyrolysis of the modified starch‐based plastic, except for copper nitrate (Cu(NO_3_)_2_) which only resulted in the formation of a black area upon scribing, though no evidence of LIG was observed. Their Raman spectra (Figure S2c, Supporting Information) show characteristic peaks resembling the PI‐LIG benchmark. However, only LIG from SP‐1.0Fe is dense enough to have reproducible results. Furthermore, Fe(NO_3_)_3_ is an abundant, cost‐effective, and less toxic material,^[^ ^49^ ^]^ and its catalytic potential has already been investigated in various forms of carbon synthesis as previously mentioned.^[^ ^37^, ^50^, ^51^, ^52^, ^53^, ^54^ ^]^ Therefore, it was selected in this study as the only additive to the SP mixture.

**Figure 2:**
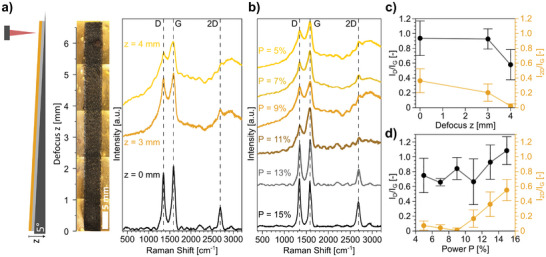
a) Graphic of the wedge used for continuous change of laser defocus *z* (left), combination of optical microscope images of SP‐1.0Fe samples at various *z* (center), and corresponding average Raman spectra taken for samples scribed with *P* = 13% at various *z* (right); b) average Raman spectra of samples scribed at a defocus of *z* = 0 mm and different *P* (5–15%); c) and d) *I*_D_/*I*_G_ and *I*_2D_/*I*_G_ ratios of the average Raman spectra reported in a) and b), respectively.

To further investigate the laser‐induced pyrolysis of the SP‐1.0Fe, a systematic optimization of laser scribing settings is necessary. A first round of optimization is carried out at a power of *P* = 13%, at different focus distances *z* = (0–4) mm. The defocus is obtained by adopting the “wedge” setup proposed by Abdulhafez et al.^[^ ^55^ ^]^ (Figure S3, Supporting Information; **Figure** **2**a), to investigate a broad span of laser fluence *H* (defined as laser power per unit area^[^ ^3^ ^]^). The wedge allows to constantly increase the focus distance of the laser along the length of the sample during scribing, and thus continuously changing the laser fluence H. Raman spectra of LIG obtained at certain defocus settings (*z* = 0, 3, 4 mm) are compared (Figure 2a,c). The transition from an amorphous carbon with broad overlapping D and G bands (*z* = 4 mm) to LIG with distinct D and G bands and a sharp 2D band (*z* = 0 mm) is highlighted. Then, by setting a fixed defocus of *z* = 0 mm, the influence of laser scribing power (range 5% < *P* < 15%) on the quality of resulting LIG is investigated by Raman spectroscopy (Figure 2b,d). A clear transition from amorphous carbon to LIG is observed as *P* increases, evidenced by the trends of *I*_D_/*I*_G_ and *I*_2D_/*I*_G_ ratios versus *P*. Overall, the best quality of LIG is obtained by scribing at *P* = 15% in focus (*z* = 0 mm). A further increase in power results in visible damage (i.e., formation of cracks and ablation) to the material.

**Figure 3:**
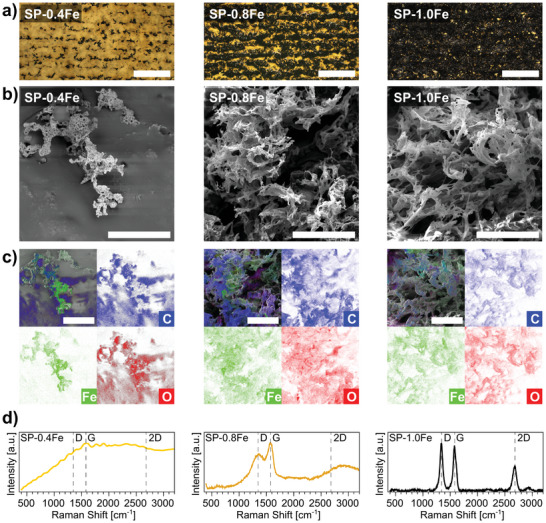
Investigation of SP‐XFe scribed with *P* = 15% and *z* = 0 mm with different concentrations of Fe(NO_3_)_3_ (columns from left to right: X = 0.4, 0.8, 1.0 mmol g^−1^): a) optical microscope images (scalebar 500 µm); b) SEM images (scalebar 50 µm); c) EDX elemental map showing C, Fe, and O (scalebar 50 µm); d) Raman spectra.

By fixing the laser power and the focus setting (*P* = 15%, *z* = 0 mm), a subsequent optimization of the concentration X of the additive Fe(NO_3_)_3_ was carried out, to find the threshold value for successful LIG formation (from 0.4 to 1.0 mmol g^−1^). The density of LIG increases with increasing Fe(NO_3_)_3_ concentration (**Figure** **3**a). The morphology of the various SP‐XFe‐derived materials formed upon laser scribing is better appreciated via SEM (Figure 3b) and corresponding EDS/EDX analyses (Figure 3c). At a low concentration, the SP‐0.4Fe substrate is decorated by small and sparse flakes of C with traces of Fe and O, as evidenced by EDS/EDX. With increasing Fe(NO_3_)_3_ concentration, the C presence on the flakes increases. The overall distribution of all three elements (C, Fe, and O) also becomes more homogeneous.

For SP‐0.4Fe, hardly any bands related to LIG appear in Raman spectra (Figure 3d), probably also due to the very low density of elemental C. For SP‐0.8Fe, the formation of amorphous carbon is observed, like in the case of low fluence (Figure 2a,b). At the highest concentration of SP‐1.0Fe, the typical bands of LIG become prominent. This indicates that a minimal concentration threshold between 0.8 and 1.0 mmol g^−1^ of Fe(NO_3_)_3_ is needed for the LIG to be created.

A comparison between LIG obtained in this work and LIG from some other bioderived and synthetic precursors is provided in SI (Table S1, Supporting Information). More extensive tables of LIG properties are reported.^[^ ^9^ ^]^

### 2.2 Microstructure and Composition

The presence of C, Fe, and O highlighted in the EDS/EDX analysis is further investigated to better understand the crystalline components of the material.

Previous studies on the pyrolysis of iron‐catalyzed biomass at high temperatures highlight the formation of Fe_3_C particles, driven by the iron‐catalytic effect of Fe(NO_3_)_3_.^[^ ^37^, ^50^, ^51^, ^52^, ^53^, ^54^ ^]^ Also in the case of LIG pyrolysis, Han et al.^[^ ^36^ ^]^ found that Fe(NO_3_)_3_‐impregnated wood resulted in a (unspecified) Fe‐C compound and elemental Fe. Dreimol et al.^[^ ^46^ ^]^ coated wood veneers with iron‐tannic acid ink before laser scribing and inferred the presence of iron carbide Fe_3_C and iron oxides Fe_x_O_y_ via XRD, but could not identify them univocally. The presence of Fe_3_O_4_ is also expected, as reported when laser scribing other iron‐containing precursors.^[^ ^56^ ^]^

**Figure 4:**
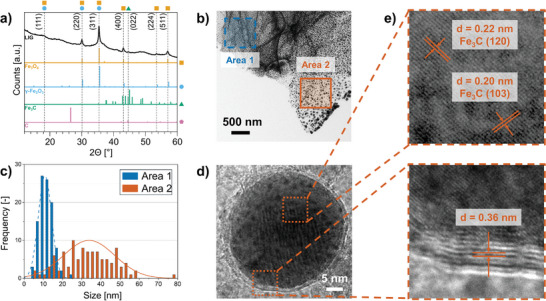
XRD and TEM analyses: a) XRD spectrum of LIG from SP‐1.0Fe (black) and theoretical peak positions for Fe_3_O_4_ (PDF#75‐0033),^[^ ^66^ ^]^ γ‐Fe_2_O_3_ (ICSD 247035),^[^ ^66^ ^]^ Fe_3_C (PDF#35‐0772)^[^ ^66^ ^]^ and graphene (C) (ICSD 7676)^[^ ^66^ ^]^ (ICSD release 2023.2); b) TEM of a LIG flake with small, densely packed nanoparticles (blue‐marked area 1), and with larger, less dense nanoparticles (orange‐marked area 2); c) particles distribution in the two different areas marked in b); d) HR TEM image of core–shell nanoparticle from the orange‐marked area 2; e) zoom of the nanoparticle's core (top), with d and orientation of crystallographic planes of Fe_3_C phase and zoom of the nanoparticle's shell (bottom), with *d* = 0.36 nm, the typical interplanar spacing of graphite.

In this work, XRD (**Figure** **4**a) shows the characteristic peaks of Fe_3_O_4_ (magnetite) / *γ‐*Fe_2_O_3_ (maghemite) structure.^[^ ^57^ ^]^ Since both compounds have the same cubic spinel structure, no clear distinction between them could be made.^[^ ^58^ ^]^ A small peak related to Fe_3_C (cementite) is detected (022) as well as other peaks that are anyway too weak to be identified and uniquely assigned. For the same reason, the presence of LIG cannot be confirmed through XRD, probably due to the low crystallinity of LIG also shown in the Raman spectrum (*I*_D_/*I*_G_ > 1).^[^ ^42^ ^]^

Therefore, TEM in combination with SAED and Mössbauer spectroscopy are used to further analyze the iron species present in the LIG. TEM confirms the presence of the Fe_3_C phase in LIG, which was not clearly observed in XRD. The low‐magnification TEM (Figure 4b) presents the morphology of a LIG flake and reveals that nanoparticles of different sizes are embedded within the LIG creating a nanocomposite. There are distinct areas with small nanoparticles and narrow distribution (blue‐marked area 1), with an average size of 10.8 nm ± 0.35 nm, and areas with larger sizes and broader distribution (orange‐marked area 2), with an average size of 33.9 nm ± 13.3 nm (Figure 4c). The discrepancy in particle size may be attributed to different origins, such as the source from varying depth layers of the scribed LIG or the distinct position in relation to the laser spot, given the existence of a temperature gradient in both cases.^[^ ^59^ ^]^ Very recently this aspect has been discussed in detail.^[^ ^60^ ^]^ It is observed that the particles have a core–shell nanostructure (Figure 4d). The core (Figure 4e, top) shows planes with interplanar spacing *d* = 0.22 nm and *d* = 0.20 nm and an interplanar angle of 78.01° (Figure S4a,b, Supporting Information). The values obtained can be assigned to the (120) and (103) lattice planes of the Fe_3_C phase and are coherent with the predicted theoretical values, calculated using cellViewer (Methods). The shell (Figure 4e, bottom) is composed of 6–7 graphitic layers with an interplanar spacing of *d* = 0.36 nm, typical of graphite.^[^ ^61^, ^62^, ^63^, ^64^ ^]^ The d values measured through the SAED (Figure S4c and Table S2, Supporting Information) are consistent with the theoretical ones. They are also in agreement with previously reported data for the Fe_3_C─C nanocomposite obtained from Fe(acac)_3_ precursor, by metal‐organic chemical vapor deposition at 700 °C.^[^ ^65^ ^]^ The authors also reported that the composition and morphology of the nanoparticles exhibit temperature‐dependent variations with the deposition range with a prevalence of Fe_3_O_4_/γ‐Fe_2_O_3_ core at 600 °C and Fe_3_C core at 700–800 °C. These findings of Fe_3_C─C core–shell nanoparticles open a new question to the whereabouts of the Fe_3_O_4_/γ‐Fe_2_O_3_ phase found with XRD.

**Figure 5:**
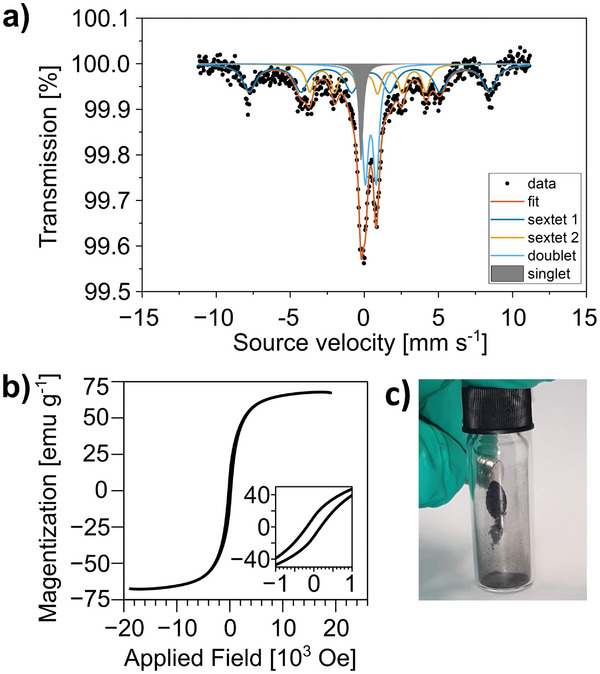
a) Mössbauer spectrum obtained at 80 K with the corresponding fit (dark orange) and deconvolution into sextet 1 (blue), sextet 2 (light orange), doublet (light blue), and singlet (grey); b) magnetization curve of SP‐1.0Fe‐derived LIG; c) picture of SP‐1.0Fe‐derived LIG powder manipulated by a magnet.

Further insight into the composition and iron phases can be obtained by studying the chemical composition of SP‐1.0Fe‐derived LIG nanocomposite using ^57^Fe Mössbauer spectroscopy. The Mössbauer spectrum obtained at *T* = 80 K (**Figure** **5**a) can be deconvoluted with four interactions: two dipolar (represented by two sextets), a quadrupole (represented by a doublet), and a monopolar interaction (a singlet). In the first sextet, the hyperfine field value of *B_hf_* = 502 kGs and the isomer shift value of *δ* = 0.38 mm s^−1^ (characteristic for Fe^3+^) indicate the presence of γ‐Fe_2_O_3_. In the second sextet, *B_hf_* = 243 kGs can be assigned to the Fe_3_C phase.^[^ ^67^, ^68^ ^]^ In the doublet, *δ* = 0.43 mm s^−1^ and the quadrupole shift Δ*Q* = 0.74 mm s^−1^ can be attributed to small γ‐Fe_2_O_3_ nanoparticles (below 10 nm, blue‐marked area 1 in Figure 4b), undergoing superparamagnetic relaxation.^[^ ^69^ ^]^ In the singlet, *δ* = −0.22 mm s^−1^ is ascribed to a non‐magnetic γ‐Fe(C) phase.^[^ ^70^ ^]^ A complete report of the spectral components obtained from the fit is given in Table S3, and a detailed discussion is provided in Supporting Information.

XPS spectra of pristine SP‐1.0Fe and LIG obtained from it (Figure S5a,b and Table S4, Supporting Information) agree with the findings of the other analyses and are discussed in Supporting Information.

Overall, these methods for investigating the crystal structure of the iron compounds show the simultaneous presence of γ‐Fe_2_O_3_, Fe_3_C, and Fe(C). While XRD gives the volumetric data, the HR TEM and SAED are local techniques that provide detailed information from a very limited area (in the order of 50 and 800 nm, respectively). Therefore, these two methods can supply complementary information for non‐homogeneous samples. The ^57^Fe Mössbauer spectroscopy, on the other hand, allows the phase analysis of iron‐containing materials by studying the electronic and magnetic state of the Fe nucleus through the hyperfine parameters with its environment, and therefore independently of whether they are in an amorphous or crystalline phase.

This information on grain size, composition, and distance between the particles is crucial for understanding the magnetic behavior, since coercive field, saturation magnetization, and remanent magnetization are functions of these parameters.^[^ ^71^ ^]^ The obtained results suggest indeed that the LIG displays soft magnetic properties.

Overall, the results of the investigations on LIG formation and microstructure highlight two main findings. First, LIG can be obtained from starch‐based bioplastic only when a metal‐based catalyst is added. Second, the obtained LIG is a magnetic nanocomposite in which core–shell nanoparticles are embedded in the porous carbon structure. These two aspects are discussed in detail in the following two sections.

### 2.3 Mechanism of iron‐catalyzed laser‐induced pyrolysis

Iron‐catalyzed graphitization of biomass with different precursor materials has been studied in literature and it has been found that Fe_3_C nanoparticles, formed in situ, are the driving catalyst for the graphitization from amorphous carbon. Depending on the system, these particles are either mobile and form graphitic nanotubes or produce core–shell structures when immobile.^[^ ^50^ ^]^ The consensus is that the iron nitrate is converted into iron oxide particles (e.g., magnetite, and wustite) which are subsequently turned into iron carbide.^[^ ^72^, ^73^ ^]^

As Dreimol et al. have previously discussed, the general process of iron‐catalyzed laser‐induced graphitization of wood should also apply to laser‐induced pyrolysis, even if on a significantly shorter timescale (seconds compared to minutes or hours). The researchers suggested that the iron oxide formation begins in the heat‐affected zone near the laser spot. However, the short timescales restrict the growth of Fe_3_C particles, resulting in the predominance of particles smaller than 20 nm.^[^ ^46^ ^]^

Since starch has a different structure than wood, which is made up of cellulose fibers and fibrils, a more detailed examination of starch‐based systems may elucidate some of the observed discrepancies. Hunter et al. investigated the influence of precursor structure on the porous carbons produced through iron‐catalyzed graphitization of biomass, comparing glucose, cellulose, and starch. The degree of crystallization in the starch sample was found to be lower than in cellulose and glucose samples, showing characteristics of turbostratic carbons. Additionally, the starch sample yielded a greater number of smaller particles than the cellulose sample, likely due to the combination of iron nitrate in solution with the starch, leading to a more homogeneous mixing. The growth of iron nanoparticles was thought to be limited by their incorporation into an amorphous carbon matrix. The researchers also discovered that the graphitization of the starch‐based carbon occurred at a slower rate than that of the other materials, allowing for better control over the resulting properties. This was attributed to the unique carbothermal reduction in the starch system, which restricted the growth of Fe_3_C particles.^[^ ^50^ ^]^

The core–shell structure of the nanoparticles can be explained based on the specific properties of the starch‐based polymer. The synthesis process involves mixing the gelatinized starch with iron nitrate, followed by vigorous stirring. This promotes the diffusion of Fe^3^⁺ ions into the amylose/amylopectin matrix. After gelation and cross‐linking, the mobility of the ions within the bioderived polymer is further hindered. Additionally, the TGA data indicate that the starch undergoes thermal decomposition at a significantly higher temperature (300 °C) compared to the decomposition of the iron nitrate into oxides (130–160 °C). This suggests that the iron oxides remain trapped within the matrix, preventing further growth.^[^ ^50^ ^]^ This finding is supported by TEM observations, which revealed that most particles were smaller than 60 nm.

The immobilization of iron nanoparticles within the starch polymer matrix, combined with the high heating rates and short timescales for laser‐induced pyrolysis, provides a plausible explanation for the observed growth of core–shell nanoparticles.

This also explains the significant presence of Fe_x_O_y_ observed in XRD, as there is not enough time for the complete conversion of iron oxide to iron carbide. Additionally, since LIG typically exhibits properties of turbostratic carbon, the slower graphitization in the starch system may be further constrained by laser‐induced pyrolysis, potentially accounting for the absence of a carbon peak in the XRD.

### 2.4 Magnetic Properties

The macroscopic magnetic properties of the powder have been assessed via VSM. The hysteresis loop (Figure 5b) of the SP‐1.0Fe‐derived LIG shows a coercive field H_c_ ≈ 200 Oe and a saturation magnetization of M_s_ ≈ 67 emu g^−1^. Such magnetic features identify our sample as a typical soft magnet.^[^ ^74^ ^]^ As such, this LIG nanocomposite powder can be manipulated with a permanent magnet (Figure 5c; Video S1, Supporting Information). Moreover, the H_c_ and M_s_ values are among the highest recorded for LIG nanocomposites (Table S5, Supporting Information). According to the Mössbauer spectroscopy results, the magnetic properties of the sample can be attributed to at least two ferro/ferrimagnetic phases represented by two sextets. A series of applications of magnetic LIG nanocomposites has already been proposed, including electromagnetic interference shielding^[^ ^13^, ^15^, ^16^ ^]^ and dye/pollutants removal for environmental remediation,^[^ ^14^ ^]^ which are viable fields of use for the presented material.

### 2.5 Starch Bioplastic Properties

**Figure 6:**
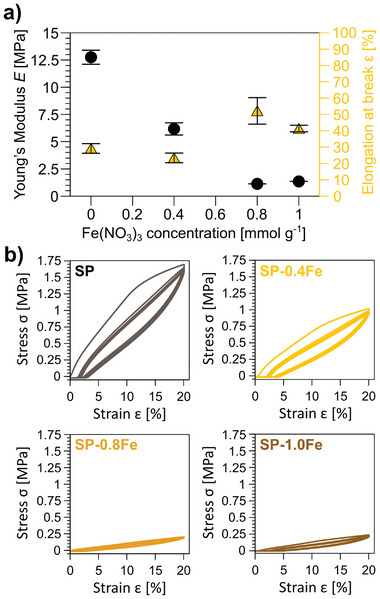
a) Young's Modulus *E* and maximum tensile elongation reached before breaking ε of SP, SP‐0.4Fe, SP‐0.8Fe, and SP‐1.0Fe; b) stress‐strain curves up to ε = 20% for SP, SP‐0.4Fe, SP‐0.8Fe, and SP‐1.0Fe.

The tensile strength of SP and SP‐XFe samples is measured to evaluate the influence of nitrate on the mechanical properties of materials. The Young's modulus and the maximum elongation reached before breaking are calculated from these measurements (**Figure** **6**a; Table S6, Supporting Information).

Samples with increasing Fe(NO_3_)_3_ concentration show a decreasing trend in Young's modulus from E ≈ 13 MPa to E ≈ 1 MPa, and a corresponding increase in maximum elongation reached before breaking. Such a trend suggests a plasticizing effect of the nitrate additive on the SP material which is in agreement with previous reports on the effect of hydrophilic inorganic salts on starch blends.^[^ ^75^ ^]^ Ions interact with the hydroxyl groups of starch chains, reducing their intermolecular hydrogen bonding and decreasing crystallinity. The hydrophilic character of salts such as Fe(NO_3_)_3_ also leads to increased water sorption of the material further fostering plasticization. Also, scribed SP‐1.0Fe samples (denoted as SP‐1.0Fe/LIG in Table S6, Supporting Information) show a similar Young's Modulus (≈0.7 MPa) but a decreased maximum elongation reached before breaking (Table S6, Supporting Information). It is to be noted that, despite the cross‐section remaining constant between pristine and scribed SP‐1.0Fe samples, part of the latter is made of LIG's highly porous structure. The mild reduction in Young's modulus may be attributed to this reason. Moreover, laser scribing induced the formation of a large number of bubbles within the substrate, which might have led to enhanced stress concentration, thus promoting breakage at lower elongation.

Looking at stress‐strain curves (Figure 6b), all the samples show a distinct viscoelastic behavior, which is consistently retained with increasing amounts of Fe(NO_3_)_3_.

**Figure 7:**
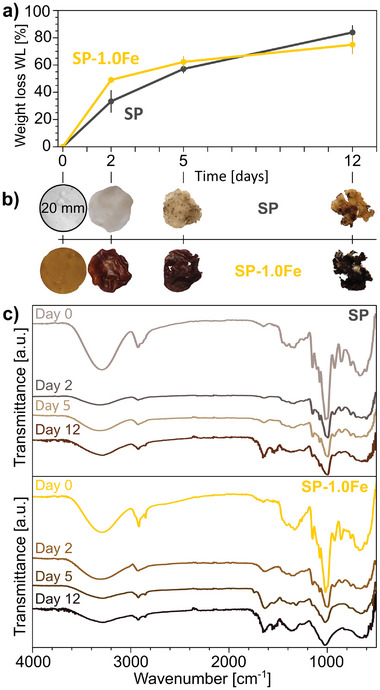
Degradation in soil of SP and SP‐1.0Fe over a time span of 12 days: a) weight loss WL over time and b) corresponding images of the degraded samples at different time points; c) FTIR spectra of samples following the degradation process.

Starch‐based bioplastics are particularly promising alternatives to traditional plastics due to the abundance, sustainability, and biodegradability of starch.^[^ ^27^ ^]^ The concept of developing a biodegradable precursor for LIG also encompasses waste management, which in this case would be the recycling of organic waste. To assess the feasibility of organic waste recycling through composting of the precursor, the degradability of the SP substrates in soil was investigated, with a focus on the effect of Fe(NO_3_)_3_ (**Figure** **7**a). Already after 2 days from the start of the test, a considerable weight loss *WL* is observed in both SP (*WL* = 33%) and SP‐1.0Fe (*WL* = 49%). When salts are added to starch, some interactions can occur between the ions in the matrix and starch molecules. For instance, high polarity ions such as nitrates have been reported to hinder hydrogen bonding between the starch molecules to a certain degree, which results in higher solubility and swelling ability of starch.^[^ ^76^ ^]^ This effect is in accordance with faster *WL* during degradation in moist soil. After 12 days of degradation, more than 80% *WL* is observed for both materials. Small variations are to be carefully considered because they could be ascribed to difficulties in the cleaning procedure of the samples due to the high degradation obtained, but the general trend remains reliable. Both SP and SP‐1.0Fe materials darkened (Figure 7b), due to soil strongly attaching to the materials’ surface and to mold formation. Both phenomena may be enhanced in the case of SP‐1.0Fe.

SEM images (Figure S6, Supporting Information) evidence an increase in roughness after 2 days, the appearance of filaments after 5 days, and the formation of slightly convex spherical granules after 12 days, as previously reported.^[^ ^30^, ^77^ ^]^

The degradability test in soil was originally planned over a 30‐day period. However, after the 12th day, a quantitative evaluation was not possible due to the small amounts of remaining materials and the above‐mentioned difficulties in their cleaning. Figure S7 (Supporting Information) reports pictures of samples of SP (left) and SP‐1.0Fe (right) retrieved on day 20. As visible, soil is strongly attached to the sample residuals, and thus no reliable information could be extracted from these samples.

Before degradation, the FTIR spectra of SP and SP‐1.0Fe (Figure 7c) show the characteristic absorption bands of starch,^[^ ^78^, ^79^ ^]^ such as C─H stretching ((2900–3000) cm^−1^), C─O, C─C and C─O─H stretching ((1100–1150) cm^−1^), and C─O─H bending ((1100–900) cm^−1^). Also, the broad absorption band in the region (3600–3000) cm^−1^ is related to OH groups on starch and glycerol, and the weak absorption at ≈1108 cm^−1^ is assigned to C─O in CHOH of glycerol.^[^ ^80^ ^]^ The pristine SP‐1.0Fe material shows two weak absorptions at ≈820 cm^−1^ and ≈800 cm^−1^ related to the nitrate ion and iron oxide.^[^ ^81^ ^]^ The spectra of the degraded materials show decreased intensities of the absorption band related to glycerol, as observed by Torres et al.,^[^ ^79^ ^]^ who reported an initial high degradation due to glycerol leaching and subsequent slower but steady *WL* associated with the biological activity present in the soil. FTIR spectra of the last withdrawals show differences in the (1150–900) cm^−1^ region as well as new complex bands in the region (1700–1450) cm^−1^ related to impurities from soil (Figure S8, Supporting Information).

### 2.6 Functional Properties of LIG

Following the initial concept of a stretchable conductor based on LIG and starch biopolymer, we investigated the electrical properties of the composite. However, accurate measurements of the electrical properties were not feasible due to the unstable ionic conductivity of the SP‐XFe, which was dependent on the water content. A more comprehensive analysis of this issue is provided in the Supporting Information (Figure S9 and Table S7, Supporting Information). Drying the samples provides more repeatable and reliable resistance results (in the order of 10 kΩ cm^−1^), but the samples themselves become very brittle, and therefore their use as flexible conductors is significantly reduced. Different possibilities are available to improve the stabilization of the SP‐XFe to environmental conditions,^[^ ^82^ ^]^ but these strongly depend on the target application and were not implemented in this study.

Investigating other properties of our magnetic LIG apart from the electrical ones can be interesting in view of finding new applications with respect to existing literature. Table S8 (Supporting Information) lists all key performance indicators for the magnetic LIG nanocomposites and compares them with relevant existing studies.

The wettability of scribed SP‐1.0Fe was assessed (Video S2, Supporting Information) with deionized water through a sessile drop technique over 30 s. The LIG showed a typical superhydrophilic behavior. If considering the very first frame at t_0_ = 0 s, when the droplet came in contact with the LIG surface, a contact angle of *θ*_c_ = 26.53° ± 3.50 was obtained, but it quickly dropped to zero before t_1_ = 1.5 s for all analyzed samples. A reference for the pristine SP‐1.0Fe substrate has been taken, showing a contact angle of *θ*_c_ = 63.39° at t_0_, *θ*_c_ = 59.78° at t_1_, and *θ*_c_ = 37.35° when it stabilized (end of the test).

Given the superwettability of starch‐based magnetic LIG and the growing interest in using porous materials for environmental remediation, a proof of concept of dye removal from water and magnetic separation of the suspension was demonstrated. LIG powder was suspended in a methylene blue (MB) solution for 30 min and then removed with a magnet. The degree of dye absorption by LIG was quantified through the measurement of the discoloration of the MB solution via UV‐Vis spectroscopy. Figure S10a (Supporting Information) shows the UV–vis spectra of the pristine MB solution and of the same solution after 30 min of absorption with the magnetic LIG powder. The latter exhibits a notable reduction in the absorption peak intensity at 665 nm, characteristic of MB, demonstrating the dye absorption capacity of the starch‐based LIG. Pictures of the MB solutions are reported in Figure S10b (Supporting Information), which compares the solution before (left), during (center), and after (right) the absorption and magnetic separation of the magnetic LIG. The absorption capacity of the magnetic starch‐based LIG is estimated to be *q* = 11.06 ± 0.43 mg g^−1^, which is on the lower end of values of magnetic carbon species reported in the literature, as shown in **Table** **1**. An improvement of dye absorption capacity may be possible through an optimization of the synthesis parameters and iron nitrate concentration. Indeed, the latter have a strong impact on the porosity of the LIG structure, responsible for sequestering the dye and, in general, pollutants.

**Table 1:** Comparison between the performances of magnetic carbon species in literature and this work.

Refs.	Precursor	Protocol	Carbonizing method	Particles	M_s_ [emu g^−1^]	MB Absorption Capacity [mg g^−1^]
[83]	AC	diol‐thermal decomposition process (Fe(acac)_3_)	n.a.	Fe_3_O_4_ / AC	16.5	n.a. (82.48 for RhB; 150.35 for MO)
[84]	sugarcane bagasse*	Fe(NO_3_)_3_ + calcination	microwave heating	γ‐Fe_2_O_3_ / AC	n.a.	36.14
[85]	glucose*	ammonium ferric sulfate + hydrothermal treatment	oven (N_2_)	Fe_3_O_4_ / AC NS	112.6	192.64
[86]	graphite powder	hydrothermal method (FeCl_3_)	n.a.	Fe_3_O_4_ @carbon	19.1	73.26
[87]	graphite powder	solvothermal method (FeCl_3_ + NaAc)	n.a.	Fe3O4 / GNS + MWCNTs	n.a.	65.79
[88]	polybenzoxazine + Fe(acac)_3_	precursor mixing	LIP	LIG / Fe_3_O_4_	≈2.2*	350.9
[89]	PDMS + lignin* + Fe_3_O_4_	precursor mixing	LIP	LIG / Fe_3_O_4_	n.a.	n.a. (microplastics absorption)
[90]	polyimide	–	LIP	none	non‐magnetic	926
[91]	polyimide	–	LIP	none	non‐magnetic	153.3
this work	SP* + Fe(NO_3_)_3_	precursor mixing	LIP	LIG / γ‐Fe_2_O_3_	67	10

^*^Bioderived precursors

Abbreviations: methylene blue (MB), activated carbon (AC), nanospheres (NS), not available (n.a.), saturation magnetization (M_s_), graphene nanosheets (GNS), laser‐induced pyrolysis (LIP), laser‐induced graphene (LIG), polydimethylsiloxane (PDMS), starch‐based polymer (SP).

## 3 Conclusions

This work demonstrates that corn starch bioplastic SP can be converted into a magnetic LIG nanocomposite via iron‐catalyzed laser‐induced pyrolysis with an IR laser. The investigation shows that a minimal concentration threshold between 0.8 and 1.0 mmol g^−1^ of Fe(NO_3_)_3_ is needed for the LIG to be created, as confirmed by Raman spectra. The best conditions for obtaining good quality LIG are identified as power P = 15%, speed S = 10%, points per inch PPI = 500, and focused beam condition when scribing on SP‐1.0Fe.

The findings of TGA support the hypothesis that the addition of Fe(NO_3_)_3_ leads to improved thermal stability, a crucial feature for LIG formation. Moreover, an in‐depth investigation of LIG through XRD, HR TEM, SAED, ^57^Fe Mössbauer spectroscopy, and XPS reveals the presence of different magnetic and non‐magnetic phases of iron compounds: γ‐Fe_2_O_3_, Fe_3_C, and Fe(C). Fe_3_C─C core–shell nanoparticles are found in LIG for the first time. More insight into the mechanism of iron‐catalyzed laser‐induced pyrolysis is provided, presenting the hypothesis of the initial growth of small iron oxides (Fe_x_O_y_), followed by the formation of iron carbide (Fe_3_C). The latter serves as a catalyst for carbonization. The immobilization of the nanoparticles resulted in the synthesis of a core–shell structure ranging in size from 5 to 60 nm. The formation of magnetic phases is further confirmed by VSM: LIG structures presented soft magnetic properties, with a coercive field of H_c_ ≈ 200 Oe and a saturation magnetization of M_s_ ≈ 67 emu g^−1^.

The mechanical properties of the SP without and with Fe(NO_3_)_3_ are assessed, showing a twofold role of the additive. Not only does it catalyze the laser‐induced pyrolysis, but it also acts as a plasticizer for SP, with a change of Young's modulus *E* (from ≈13 MPa to ≈1 MPa). The SP compound is highly degradable in soil after 12 days of burial and its weight loss is not affected by the addition of Fe(NO_3_)_3_. The LIG obtained from SP‐1.0Fe is highly hydrophilic, as assessed by contact angle measurements (*θ*_c_ = 0° within 1.5 s from the placement of a water droplet).

Overall, this study reveals that starch is a bioderived and degradable LIG precursor and represents a cost‐effective and abundant alternative to synthetic polymers. The material's characterization highlights that the direct use of SP‐XFe as a substrate for flexible “green” electronics is, unfortunately, hampered by its large hygroscopicity and ionic conductivity. However, starch‐derived LIG can still be adopted in combination with other substrates, including bioderived ones. For example, starch‐derived LIG in the form of powder can be mixed with various dispersing media (e.g., solvents, pastes) to create inks or paints and thus applied as a conductive coating on different substrates. Alternatively, and like the approaches proposed in,^[^ ^46^, ^50^, ^60^ ^]^ the SP itself can be used as a coating and then laser‐scribed to obtain LIG. Moreover, the serendipitous discovery of complex nanostructures and their magnetic properties shifted the focus to a more innovative and less explored path, allowing us to present a bioderived soft magnetic LIG nanocomposite for the first time. This process represents a bioderived alternative to obtain carbon/iron composites for environmental remediation, such as absorption of organic pollutants, dye removal, and wastewater treatment. Indeed, the laser process represents an original approach for the production of such nanocomposites in comparison to already published systems, which were produced with more conventional oven pyrolysis. A proof of concept for dye absorption in wastewater and the magnetic separation of the LIG suspension was demonstrated, showing that the absorption capacitance of the unoptimized starch‐based LIG is ≈10 mg g^−1^. Future studies dedicated to this application should focus on investigating how the SP synthesis and laser processing parameters influence the porosity of LIG, and hence its absorption capacity.

## 4 Experimental Section

### Materials

Commercially available food‐grade corn starch (Maizena by Unilever plc) was obtained from a local supermarket. Analytical grade glycerol plasticizer (>99.5%), glacial acetic acid (>99%), iron (III) nitrate nonahydrate (Fe(NO_3_)_3_·9H_2_O), chromium (III) nitrate nonahydrate (Cr(NO_3_)_3_·9H_2_O), nickel (II) nitrate hexahydrate (Ni(NO_3_)_2_·6H_2_O), cobalt (II) nitrate hexahydrate (Co(NO_3_)_2_·6H_2_O), copper (II) nitrate trihydrate (Cu(NO_3_)_2_·3H_2_O), iron (III) chloride (FeCl_3_) (97%) and methylene blue dye were purchased from Sigma‐Aldrich.

### Starch Bioplastic Fabrication Process

For SP fabrication, glycerol (3.15 g) and acetic acid (2.5 g) were dissolved into deionized water (30 ml). Corn starch (5 g) was added to the solution, and the mixture was heated at 150 °C with a hotplate. The process was performed under continuous mechanical stirring at 150 rpm (Heidolph RZR‐2000). The heating was carried out until the starch mixture reached a waxy texture, indicating the completion of the gelatinization process. The mixture was then cooled down at room temperature (RT) and cast onto a silicone sheet using a rectangular‐shaped plexiglass mold (length 75 mm, width 25 mm, thickness 2 mm). The formed films underwent a 72‐hour drying process under ambient conditions.

For SP‐XFe fabrication (and in general when adding metal salts), an analogous approach was adopted. Separate batches of starch mixtures were prepared using the SP formulation and method described above. After the starch mixtures cooled down to RT, varying concentrations of iron (III) nitrate salt (0.4, 0.8, and 1.0 mmol g^−1^; denoted as SP‐XFe, with X being the concentration) were incorporated into each batch through mechanical mixing at 100 rpm (Heidolph RZR‐2000), until homogeneous mixtures were achieved. The cooled mixtures were cast onto silicone sheets using the above‐mentioned molds and underwent a 72‐hour drying period under ambient conditions. The SP was mixed with other metal salts in the same manner, using a concentration of 1.0 mmol g^−1^ unless otherwise specified.

### Laser Scribing

A CO_2_ laser cutter/engraver (Universal Laser Systems VLS 3.50, *P_max_* = 50 W, laser emission wavelength *λ*  =  10.6 µm) equipped with a 2.0 beam collimator (nominal beam size 130 µm) was used to create LIG patterns on all tested materials. The laser scribing was carried out in ambient conditions, with films of average thickness of 0.25 mm attached to a glass slide. The laser cutter was operated in raster mode, with laser settings: power *P* = 5–15%, speed *S* = 10%, points per inch *PPI* = 500, image density *ID* = 5 (defining a spacing between consecutive raster lines of 280 µm) and positive defocus *z* = (0–4) mm. A sweep over a continuous defocus was achieved by using a 3D printed wedge/sample holder with a slope of 5°, which could accommodate samples with size 75 × 25 mm^2^ (Figure 2a and Figure S3, Supporting Information).

### Microscopic Characterization

LIG morphology was investigated with a Hirox HR 5000 (E) digital optical microscope equipped with a High‐Range Motorized Triple Zoom Lens, and with a Phenom XL SEM (ThermoFisher Scientific) equipped with EDS/EDX and operating at 10 kV. Non‐conductive samples were coated with a 10 nm‐thick Au–Pd layer using a sputter‐coater (Quorum), for enabling SEM imaging.

### Raman Spectroscopy

The Raman spectroscopy was carried out with a LabRAM HR Evolution Raman microscope at a wavelength of *λ_i_* = 532 nm, with a power percentage of 3.2% (nominal power = 3.2 mW). An integration time of 3 s per 15 accumulations, a slit size of 300 µm, and a ×100_VIS objective were used. Multiple single‐point spectra were taken at different locations on each sample. The obtained raw data were then post‐processed with peak‐conservative smoothing, baseline correction, and averaged (over at least three samples). The peak ratios *I*_D_/*I*_G_ and *I*_2D_/*I*_G_ were obtained from the post‐processed spectra, but the plots came from the raw data (apart from Figure 1b,c, in which post‐processed spectra were displayed).

The *I*_D_/*I*_G_ ratio was used to evaluate the crystalline size, according to Equation 1.^[^ ^44^ ^]^

Equation 1. Crystalline size formula.

(1) $$L_{a}\left(\mathrm{nm}\right)=\left(2.4\cdot 10^{-10}\right)\lambda_{i}^{4}{\left(\frac{I_{D}}{I_{G}}\right)}^{-1}$$

### TGA

The thermal stability of SP and SP‐XFe samples was determined with a thermogravimetric analyzer (Perkin Elmer TGA 4000). The temperature scans were carried out with 5340 points in the range 30 < T < 900 °C, at a rate of 10 °C min^−1^ and including a pause of 2 min at 900 °C at the scan end, under air and nitrogen environment.

### XRD

XRD measurements were performed in *2Θ* = (10–60)° with a Panalytical Empyrean diffractometer (PANalytical, the Netherlands, Cu tube, *λ* = 1.5418 Å) on scraped‐off LIG powder from SP‐1.0Fe. The peaks for the comparison with the expected materials were generated using the Inorganic Crystal Structure Database (ICSD), PowderCell, and DIFFRAC.EVA.

### TEM

TEM analyses were conducted using the ThermoFisher Tecnai TF 20 X‐TWIN microscope (200 kV), equipped with an Eagle 2k HR camera and a Field Emission Gun. Scraped‐off LIG powder samples were directly drop‐casted from an ethanol solution onto the Cu TEM grid. The histograms were generated based on a dataset of 100 nanoparticles. The HR TEM images underwent Fast Fourier Transform and ImageJ software was used for post‐processing to determine the value of interplanar spacing d and interplanar angle. SAED images were obtained with an 800 nm diameter aperture. The ring diffraction was indexed and compared with theoretical values using cellViewer by Crystallographic Tool Box (CrysTBox) software. For Fe_3_C, the Fe_3_C Crystallographic Information File was used [Materials Data on Fe_3_C by Materials Project10.17188/1263035].^[^ ^92^ ^]^

### Mössbauer Spectroscopy

Scraped‐off LIG powder samples were used. The ^57^Fe Mössbauer spectrum of the sample was recorded at *T* = 80 K in a transmission mode using a ^57^Co(Rh) source with an activity of ≈25 mCi for 14.4 keV γ‐rays w RENON MsAa‐4 spectrometer. The spectrum was recorded in a 1024‐channel analyzer and folded to 512 channels for analysis. The Mössbauer spectrum was fitted by least squares procedure using homemade PMos software using a Lorenzian profile in the thin absorber approximation. The acquisition time of the spectrum was two weeks.

### XPS

The XPS analyses were carried out in a Scanning XPS system (PHI VersaProbeII) using monochromatic Al Kα (1486.6 eV) X‐rays focused to a 100 µm spot and scanned over an area of 400 µm x 400 µm. The photoelectron take‐off angle was 45° and the pass energy in the analyzer was set to 117.50 eV (0.5 eV step) for survey scans and 46.95 eV (0.1 eV step) to obtain high energy resolution spectra of Fe 2p3/2, C 1s, O 1s, N 1s, and Si 2p regions.

A dual beam charge compensation with 7 eV Ar^+^ ions and 1 eV electrons was used to maintain a constant sample surface potential regardless of the sample conductivity. All XPS spectra were charge referenced to the unfunctionalized, saturated carbon (C─C) C1s peak at 285.0 eV. The operating pressure in the analytical chamber was <3 × 10^−9^ mbar. Deconvolution of spectra was carried out using PHI MultiPak software (v.9.9.3). Spectrum background was subtracted using the Shirley method.

### VSM

The magnetization profile of the LIG was characterized by a Vibrating‐Sample Magnetometer (MicroSense, EZ9 USA). The SP‐1.0Fe‐derived LIG was scraped from the substrate and the powder (mass: 1.4 mg) was enclosed in a 3D‐printed cylindrical container (*⌀*_int_ = 4 mm, *h_int_* = 1 mm). The container was mounted on the instrument's sample holder. A magnetic field H was applied in the orthogonal direction to the sample and varied in the range −1.9 < H < 1.9 T, at a step of 0.01 T from 0 to ±0.5 T, and at a step of 0.02 T from ±0.5 T to ±1.9 T. The frequency of sample vibration was 75 Hz. The magnetization hysteresis loop was obtained after subtracting the data from the empty container and the values were normalized by the weight of the LIG powder.

### Mechanical Testing

An Instron 5965 series universal testing machine equipped with a 10 N load cell was employed for mechanical properties assessment, coherently with ASTM D882 standards. The testing protocol included four blocks with increasing maximum strains *ɛ*_max_ (10%, 20%, 30%, and 40%), each constituted of five consecutive cycles and a pause of 20 s at the end. The elongation rate was set to 50 mm min^−1^. At the end of the cyclic protocol, a single tensile elongation at break was carried out at 50 mm min^−1^. The elongation at break values were evaluated as the maximum elongation reached before breaking.

### Precursor Degradation in Soil

Tests of materials degradability in soil were conducted by burying samples in soil and evaluating their weight loss WL at different time points over a 30‐day period at RT. The tests were carried out in polypropylene pots filled with all‐purpose soil obtained from the local market (200 g). For every time point, a set of three samples, each with a 20 mm diameter and 0.25 mm thickness (average weight of 117 mg for SP and 92 mg for SP‐1.0Fe), were weighed, freeze‐dried at −80 °C for 48 h, and weighed again. Such dry weights were accounted for as W_0_. The sets were then buried in the pots at a soil depth of 50 mm from the pot top, which had been previously moisturized with tap water (20 ml), and then covered with a punched aluminum foil.

At each time point, samples were unburied and gently cleaned by brushing and rinsing with deionized water to remove soil residuals, then freeze‐dried at −80 °C for 48 h and weighed (W_t_). The WL was calculated according to Equation 2.

Equation 2. Weight loss formula.

(2) $$WL\left(\%\right)=\frac{W_{0}-W_{t}}{W_{0}}\cdot 100$$

To visually inspect the effects of degradation, SEM images and FTIR spectra were taken at each time point, providing insights into the structural changes that occurred during soil burial.

### FTIR Spectroscopy

FTIR spectra of the materials were recorded with a Shimadzu IR Affinity‐1 instrument equipped with a universal ATR (Attenuated Total Reflection) accessory (MIRacle 10). The sample was uniformly pressed against the crystal surface using a spring‐loaded anvil. Mid‐IR spectra were obtained by averaging 64 scans over the range 4000–600 cm^−1^ at 4 cm^−1^ resolution.

### Resistance Measurements

An ESPEC 242 SH climatic chamber was used to perform the resistance measurements in controlled environments on pristine and scribed SP‐1.0Fe samples (dimensions 40 mm length and 5 mm width). The process was carried out at room temperature (25 °C) and relative humidity from 90% to 30% with a step of 30%. At each set humidity level, the samples were stabilized. The resistance values were then measured with a Keithley 2604B source meter and acquired with a Python script.

### Wettability

Contact angle measurements were conducted with a Biolin Theta Flex Optical tensiometer provided with a video camera, a manual XYZ sample stage, an automatic dispenser unit, and an LED light source. Using the sessile drop technique, a drop of deionized water (*V* = 5 µl) was dispensed on the surface of interest and recorded for 30 s. The calculation of the contact angle was performed with the One Attension software. Measurements were conducted at room temperature. Three samples of scribed SP‐1.0Fe were tested and averaged. Videos were recorded of the tests, to prove the fast water absorption. One sample of pristine SP‐1.0Fe was taken as a reference.

### Dye Absorption

Under UV‐blocking light, MB dye was dissolved in deionized water at a concentration of *C*_0_ = 8 mg l^−1^. The magnetic LIG powder (2 mg) and the MB solution (4 ml) were added to a glass ampoule. The mixture was magnetically stirred at 500 rpm for 30 min (absorption period) at room temperature. After the absorption period, the magnetic LIG powder was separated from the solution using a magnet placed at the bottom of the ampoule, and the solution was transferred to cuvettes. UV‐Vis spectra (Shimadzu UV‐18000) were recorded for the MB solution before and after absorption and separation of the magnetic LIG powder. The concentration of the MB solution after absorption *C_e_* was calculated using a calibration curve. This was a standard linear regression function obtained from data of optical absorbance *A* (in a.u.) at 665 nm for a series of MB solutions with known concentrations *C* (in the range 1–8 mg l^−1^). The function was defined as *A* = 0.2134 *C* (with *R^2^* = 0.9987). The absorption capacitance *q* was calculated according to Equation 3, where *C*_0_ and *C*_e_ were the concentration (in mg l^−1^) before and after the absorption, *V* was the solution volume (in l) and *M* was the mass of used LIG (in g).

Equation 3. Absorption capacitance formula.

(3) $$q=\frac{\left(C_{0}-C_{e}\right)\cdot V}{M}$$

## Conflict of Interest

The authors declare no conflict of interest.

## Supporting information



Supporting Information

Supplemental Video 1

Supplemental Video 2

## Data Availability

The data that support the findings of this study are available from the corresponding author upon reasonable request.
