# Supplementary material for: From Corn Starch to Nanostructured Magnetic Laser‐Induced Graphene Nanocomposite
Source: Small. 2024 Oct 18;20(52):2405252. doi: 10.1002/smll.202405252 (PMC11673402; doi:10.1002/smll.202405252)
Supplement: Supplementary file 1 — Supporting Information [file SMLL-20-2405252-s002.pdf]

NANO · MICRO
small

Supporting Information

for *Small*, DOI 10.1002/smll.202405252

From Corn Starch to Nanostructured Magnetic Laser-Induced Graphene Nanocomposite

*Sreenadh Thaikkattu Sankaran, Alexander Dallinger, Anna Chiara Bressi, Attilio Marino, Gianni Ciofani, Aleksandra Szkudlarek, Vitaliy Bilovol, Krystian Sokolowski, Birgit Kunert, Hana Kristin Hampel, Hilda Gomez Bernal and Francesco Greco**

From Corn Starch to Nanostructured Magnetic Laser-Induced Graphene Nanocomposite

Sreenadh Thaikkattu Sankaran[1,2,§], *Alexander Dallinger*[3,§], *Anna Chiara Bressi*[1,2 §], *Attilio Marino*[4], *Gianni Ciofani*[4], *Aleksandra Szkudlarek*[5], *Vitaliy Bilovol*[5], *Krystian Sokołowski*[5], *Birgit Kunert*[3], *Hana Kristin Hampel*[6], *Hilda Gomez Bernal*[1], *Francesco Greco*[1,2,3,7,]*

[1] The Biorobotics Institute, Scuola Superiore Sant'Anna, Viale Rinaldo Piaggio 34, 56025 Pontedera, Italy.

[2] Department of Excellence in Robotics & AI, Scuola Superiore Sant'Anna, Piazza Martiri della Libertà 33, 56127 Pisa, Italy.

[3] Institute of Solid State Physics, NAWI Graz, Graz University of Technology, Petersgasse 16, 8010 Graz, Austria.

[4] Smart Bio-Interfaces, Istituto Italiano di Tecnologia, Viale Rinaldo Piaggio 34, 56025 Pontedera, Italy.

[5] Academic Centre for Materials and Nanotechnology, AGH University of Krakow, av. Mickiewicza 30, 30-059 Krakow, Poland.

[6] Institute of Experimental Physics, NAWI Graz, Graz University of Technology, Petersgasse 16, 8010 Graz, Austria.

[7] Interdisciplinary Center on Sustainability and Climate, Scuola Superiore Sant'Anna, Piazza Martiri della Libertà 33, 56127 Pisa, Italy.

[§] These authors contributed equally to this work.

* Corresponding author: Francesco Greco francesco.greco@santannapisa.it

Starch bioplastic manufacturing

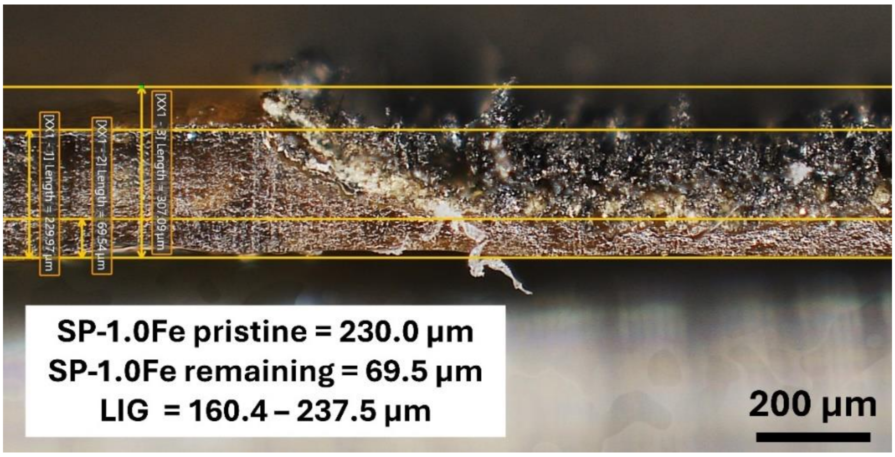

Figure S1. Cross-section of a scribed SP-1.0Fe sample and estimation of LIG thickness.

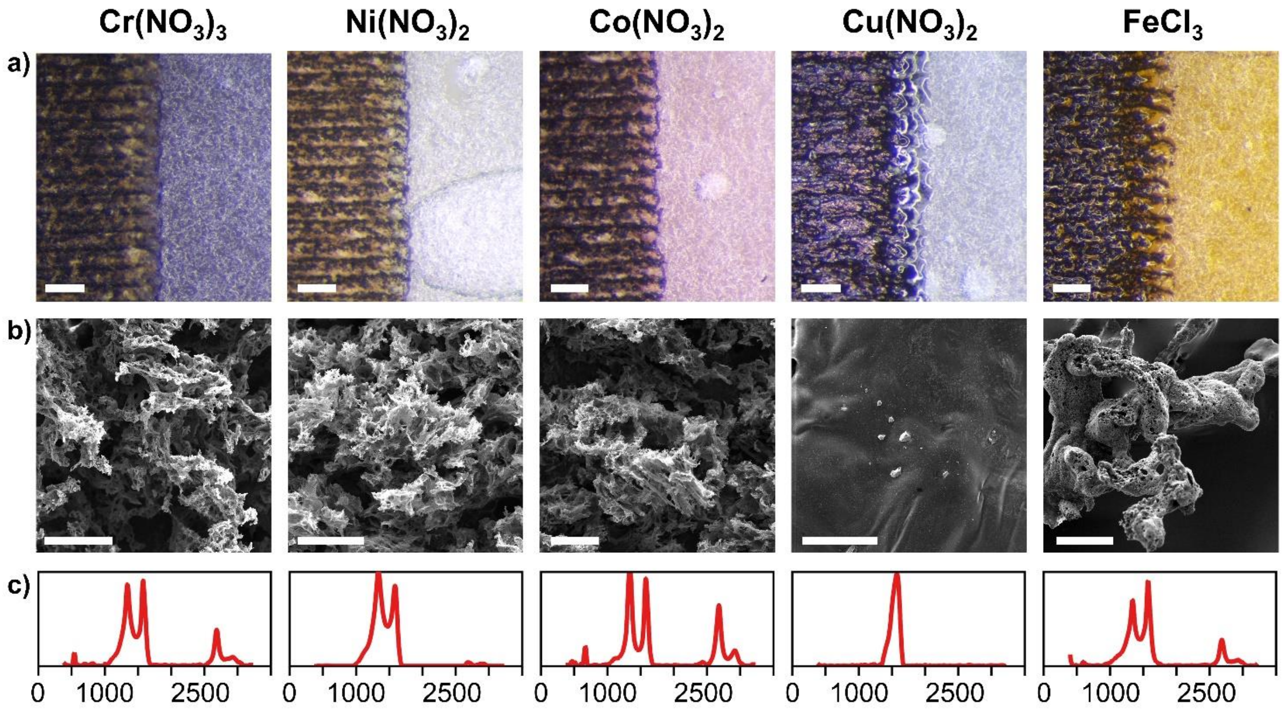

Figure S2. a) Optical microscope images of SP with 1 mmol g^{-1} of $Cr(NO_3)_3$, $Ni(NO_3)_2$, $Co(NO_3)_2$, $Cu(NO_3)_2$, $FeCl_3$, laser-scribed (left) and pristine (right). The color mismatch between the pristine SP samples observed under the optical microscope and with direct human eyesight may be caused by metamerism (scalebar 200 µm); b) SEM images of SP with $Cr(NO_3)_3$, $Ni(NO_3)_2$, $Co(NO_3)_2$, $Cu(NO_3)_2$, $FeCl_3$ (scalebar 50 µm); c) Raman spectra of scribed SP with $Cr(NO_3)_3$, $Ni(NO_3)_2$, $Co(NO_3)_2$, $Cu(NO_3)_2$, $FeCl_3$.

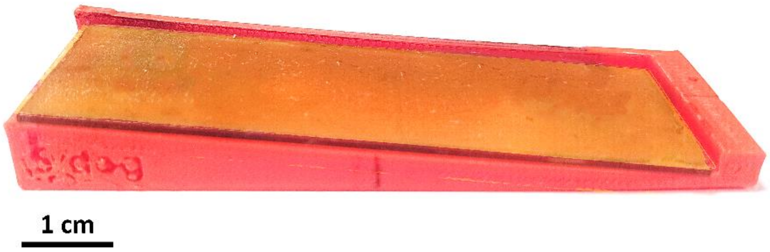

Figure S3. Picture of the “wedge” set up for defocused laser scribing, as previously presented in [1].

Raman Comparison

Table S1. Comparison between LIG obtained in this work and LIG from other bioderived and synthetic precursors.

Ref.	Precursor	I_D/I_G	I_{2D}/I_G
[2]	Wood	0.25-0.8	0.5-0.74
[3]	Coconut shell<br>Potato skin	≈0.8*	≈0.7*
[4]	Leaves	≈0.8*	≈0.4*
[5]	Bamboo	0.7	≈0.5*
[6]	Agglomerated cork	0.2	0.5
[7]	Natural cork	0.6	0.5
[8]	Paperboard	0.71	≈0.5*
[9]	Filter paper	0.5	≈0.4*
[10]	Cellulose nanocrystals	≈1.2*	≈0.4*
[11]	Carboxymethyl Xylan (hemicellulose)	≈0.8*	≈0.5*
[12]	Cellulose nanofibers	≈1.5*	≈0.2*
[13]	Kraft lignin + poly(vinyl alcohol)	0.39	≈0.5*
[14]	Nano kraft lignin + cellulose nanofibers	≈0.9*	≈0.2*
[15]	lignosulfonate + poly(vinyl alcohol), urea	0.33	≈0.5*
[16]	Polyimide	1.2	0.7
[17]	Poly(ether–ether–ketone) + Polydimethylsiloxane	≈0.5*	≈0.9*
This work	Starch bioplastic + iron nitrate	1.1	0.6

HR TEM and SAED

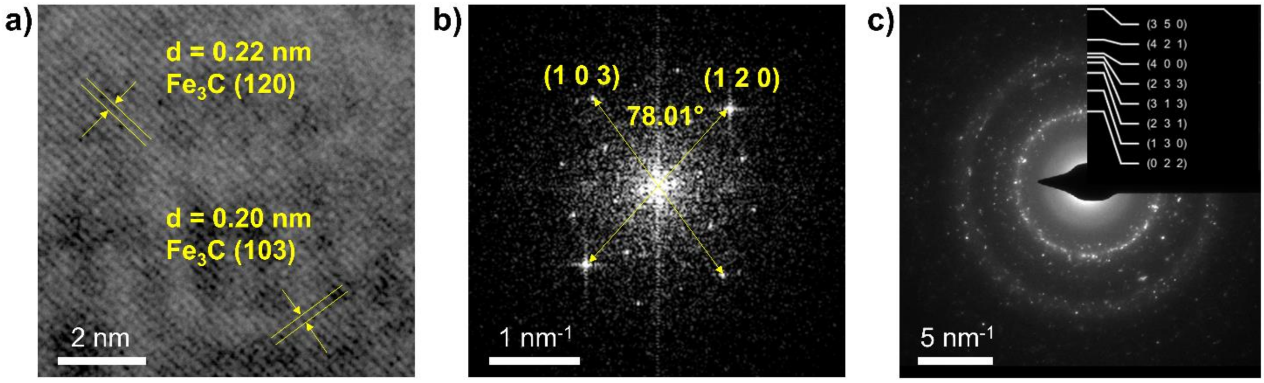

Figure S4. HR TEM and SAED analyses: a) HR TEM image of Fe_3C nanoparticle's core used for Fast Fourier Transform (FFT) with ImageJ software, with interplanar spacing d and orientation of crystallographic planes; b) results of FFT, with crystallographic lattice planes' indices and interplanar angle; c) ring diffraction obtained with SAED and indexed with CrysTBox software.

Table S2. Theoretical and measured values for radii of diffraction rings for Fe_3C phase, interplanar spacing d, and angle of diffraction Θ, calculated with Bragg's law (with λ= 0.15418 nm, as stated in Methods).

**Radius [nm^{-1}]**		**d [nm]**		**2Θ [°]**	
Theoretical	*Measured*	*Theoretical*	*Measured*	*Theoretical*	*Measured*
4.934	4.896	0.203	0.204	44.64	44.41
6.309	6.312	0.159	0.158	58.00	58.41
7.532	7.53	0.133	0.133	70.85	70.85
8.241	8.226	0.121	0.122	79.15	78.38
8.625	8.599	0.116	0.116	83.30	83.30
8.861	8.847	0.113	0.113	86.03	86.03
9.809	9.792	0.102	0.102	98.19	98.19
11.878	11.879	0.084	0.084	133.20	133.20

Mössbauer Spectroscopy

The Mössbauer spectrum obtained at 80 K with the corresponding fit (dark orange) and deconvolution into sextet 1 (blue), sextet 2 (light orange), doublet (light blue) and singlet (grey) is reported in **Figure 5a**.

- Sextet 1: the hyperfine field above 500 kGs allows us to conclude that the phase is iron oxide. The isomer shift value is typical for Fe^{3+} and suggests γ-Fe_2O_3 phase (maghemite, Fe^{3+} only).[18] Nevertheless, the line width is rather wide (0.54 mm s^{-1}). We could not reject that might be partial contribution coming from Fe_3O_4 (magnetite, a mixture of Fe^{3+} and Fe^{2+})[19], which possesses a similar hyperfield value. Magnetite has a rather complex spectrum at this temperature and contributions coming from the subspectra corresponding to eventual Fe^{2+} ions ($\delta \approx 0.7$- 0.8 mm s^{-1}) might be masked by the other subspectra from Fe^{3+} ions of magnetite and/or as well as by the spectrum of maghemite phase. All this may explain the relatively wide line width of Sextet 1, which could be in fact, a simplified representation of γ-Fe_2O_3/Fe_3O_4.[20] It is well known that any of these phases provides a high saturation magnetization and a low coercive field (both are magnetically soft materials).[19], [21]
- Sextet 2: the hyperfine field value does not originate from iron oxide and the isomer shift value (typical for Fe^{3+}) can be assigned to the Fe_3C phase (also called cementite)[22], [23], which is generally found in similar systems[20], [24]. Its high magnetization saturation value (above 100 emu g^{-1} for nanoparticles[25]) can also contribute to the magnetic response of the whole sample as was measured by VSM as discussed later.
- Doublet: it corresponds to a non-magnetic Fe^{3+} - containing phase. The quadrupole splitting of approximately 0.74 mm s^{-1} with the obtained isomer shift value could be attributed to small γ-Fe_2O_3 nanoparticles (below 10 nm, see Fig. TEM1 b)) undergoing superparamagnetic (SPM) relaxation.[26] The magnitude of the isomer shift allows us,

without a doubt, to discard the existence of Fe_3O_4 in this case. It may be an argument in favor of that Sextet 1 also, most likely, is only γ-Fe_2O_3 rather than γ-Fe_2O_3/Fe_3O_4 mixture. It is worth mentioning that at such a low temperature of 80 K, these nanoparticles are still relaxed. This is possible because they are well separated in the organic matrix and do not interact with each other. This causes the blocking temperature of this set of nanoparticles to shift to even lower temperatures. Similar phenomenon on γ-Fe_2O_3 system of nanoparticles has been observed with zeolite as a matrix.[27]

- Singlet: the value of the isomer shift allows us to assign it to a non-magnetic γ-Fe(C). This phase has often been reported to be present in syntheses containing iron and carbon.[20], [28], [29] Probably due to the low content of γ-Fe(C) or/and the small size of its particles, it is not clearly observed in the XRD pattern.

Table S3. Hyperfine parameters for Mössbauer spectroscopy results: B_{hf} is the hyperfine field (± 0.5 kGs), δ is the isomer shift (± 0.01 mm s^{-1}) relative to the ^{57}Co(Rh) source at room temperature, ΔQ is the quadrupole shift (± 0.01 mm s^{-1}), ε_Q is the quadrupole splitting (± 0.01 mm s^{-1}), A is the area (with a relative error of less than 10 %), and Γ_a is the full width at a half maximum of absorption lines (± 0.01 mm s^{-1}).

**Interaction**	**B_{hf} (kGs)**	**δ (mm s^{-1})**	**ΔQ (mm s^{-1})**	**ε_Q (mm s^{-1})**	**A (%)**	**Γ_a (mm s^{-1})**
Sextet 1	502.0	0.38	-	- 0.09	41.0	0.54
Sextet 2	242.9	0.26	-	- 0.03	24.2	0.32
Doublet	-	0.43	0.74	-	26.4	0.26
Singlet	-	- 0.22	-	-	8.4	0.18

XPS

Surface concentrations of chemical bonds obtained from fitting acquired XPS core-level spectra for SP-1.0Fe (**Figure S5a)** and LIG from SP-1.0Fe (**Figure S5b)** are listed in **Table S4**. C 1s, N 1s, O 1s, Si 2p and Fe 2p3/2 were registered for both samples and analyzed in details:

- C 1s: they were well-fitted using four components. The first line (285.0 eV) showed a symmetric and narrow shape, pointing out the existence of sp^2 and sp^3 – bonded carbon and/or carbide fractions in the samples. The second line (286.6 eV) indicated the presence of C-O and/or C-OH and/or C-O-C bonds. The third line (288.2 eV) indicated the presence of C=O and/or O-C-O species. The fourth line (289.1 eV) came from either O-C=O and/or N-C=O groups.[30], [31], [32]
- N 1s: they were fitted with three lines. The first line (400.4 eV) indicated the presence of N-C=O type bonds. The second line (401.9 eV) originated from NH_4^+ type ions. The third broad line (407.2 eV) came from nitrate NO_3^- compound, most probably $Fe(NO_3)_3$.[32], [33]
- O 1s: they were fitted using three components. The first line (530.9 eV) indicated the presence of metal oxide (O-Fe). The second line (532.5 eV) indicated the presence of defective oxygen in metal oxides and/or O-Si and/or O-N and/or O=C groups. The third line (533.4 eV) could originate either from O-H and/or C-O type bonds and/or adsorbed H_2O.[33], [34]
- Si 2p: they were fitted with doublet structure (doublet separation $p_{3/2} – p_{1/2}$ equals 0.6 eV). The main $2p_{3/2}$ line (102.2 eV) indicated the presence of C-Si-O type bonds like in silicones/siloxanes.[33], [35]
- Fe $2p_{3/2}$: for both samples, there were significantly multiplet splitting characteristics for high-spin Fe^{3+} compounds. Each Fe $2p_{3/2}$ spectrum was fitted with up to eight components. The first line (707.8 eV) indicated the presence of metallic and/or carbide iron. The second line (709.6 eV) indicated the Fe^{3+} in iron oxide Fe_2O_3. Six lines within the energy range of 710-716 eV were due to the multiplet splitting phenomena.[34], [36], [37]

Figure S5. XPS spectra of a) SP-1.0Fe and b) LIG from SP-1.0Fe samples. High-resolution XPS spectra and peak deconvolutions of C 1s, N 1s, O 1s, Si p, and Fe 2p3/2 are reported below for both samples.

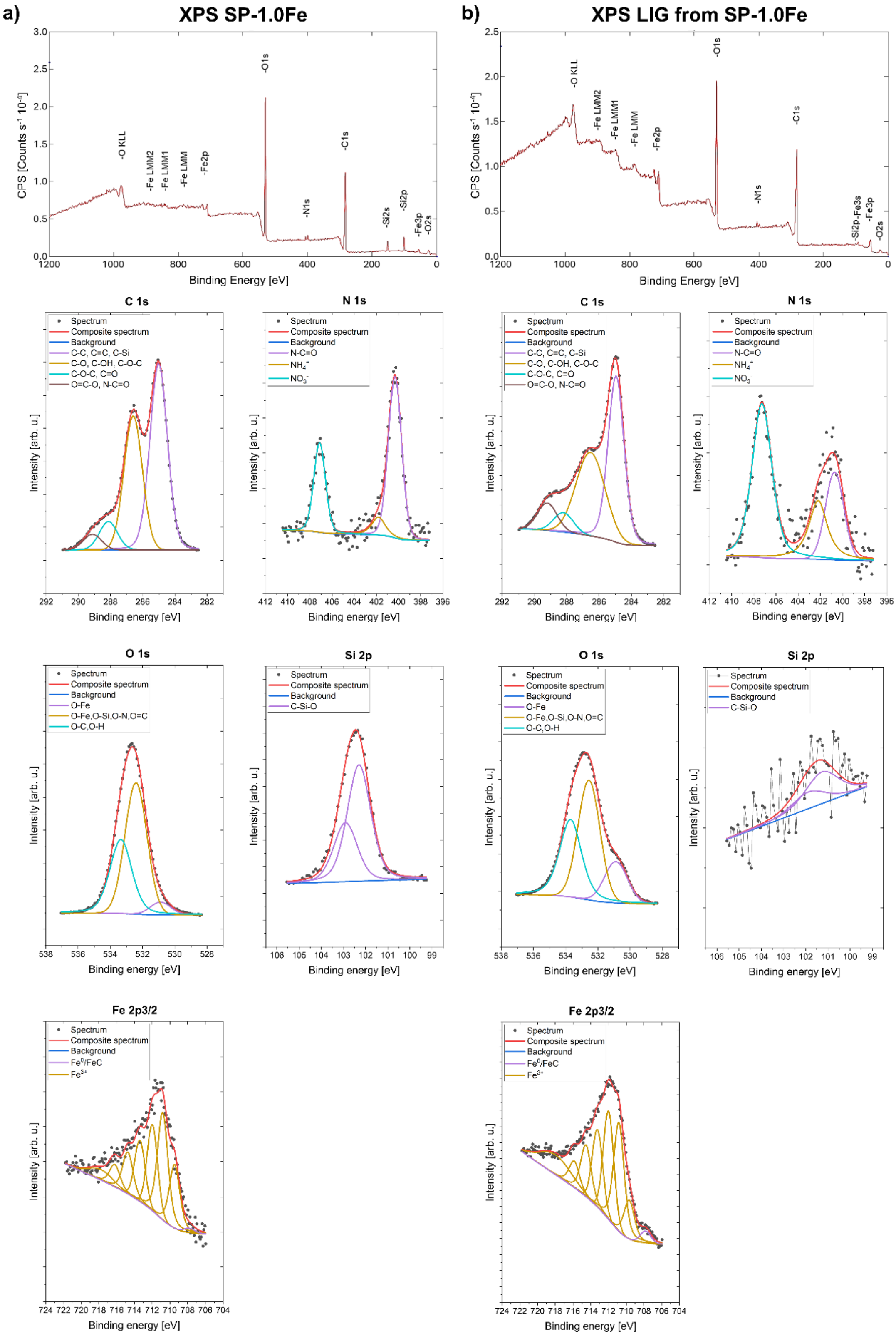

Table S4. Surface composition (atomic %) of SP-1.0Fe and LIG from SP-1.0Fe samples determined by XPS measurements.

Element	C				N			O			Si	Fe	
Energy [eV]	285.0	286.6	288.2	289.1	400.4	401.9	407.2	530.9	532.5	533.4	102.2	707.8	709.6
Sample/Groups	C-C C=C C-Si	C-O, C-OH, C-O-C	C-O-C. C=O	O=C-O N-C=O	N-C=O	NH_4^+	NO_3^-	O-Fe	O-Fe, O-Si, O-N, O=C	O-C, O-H	C-Si-O	Fe^0/FeC	Fe^{3+}
SP-1.0Fe	28.5	20.3	4.3	2.3	1.6	0.2	0.8	1.9	20.2	12.9	5.1	0.1	1.9
LIG from SP-1.0Fe	26.4	22.2	3.5	4.9	0.7	0.7	1.6	5.9	16.4	12.5	0.2	0.2	4.9

Magnetic properties

Table S5. Vibrating-Sample Magnetometer values in the existing literature of LIG compared to the current work.

Particles	Saturation Magnetization M_s [emu g^{-1}]	Coercive Field H_c [Oe]	Reference
LIG/Fe_3O_4	5.27	74	[38]
LIG/Ni	30.3	n.a.	[39]
LIG/Fe_3O_4	14.4	299	[40]
LIG/FeCo	36.3	310	[40]
LIG/Fe_3O_4	≈2.2*	≈350*	[41]
LIG/γ-Fe_2O_3	67	200	this work

*extracted from the graph

Starch bioplastic properties

Table S6. Young's Modulus and tensile elongation at break of non-scribed and scribed SP-XFe.

Sample	Youngs Modulus [MPa]	Elongation at break [%]
SP	12.8 ± 0.7	27 ± 3
SP-0.4Fe	6.2 ± 0.6	22 ± 3
SP-0.8Fe	1.1 ± 0.1	50 ± 8
SP-1.0Fe	1.4 ± 0.1	41 ± 2
SP-1.0Fe/LIG	0.7 ± 0.1	27 ± 3

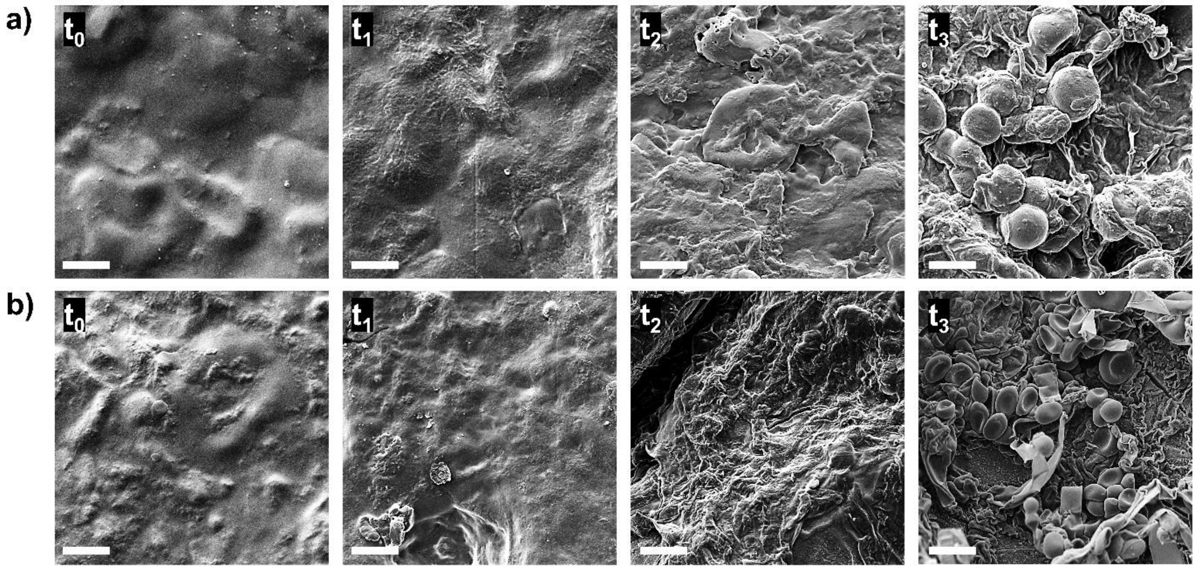

Figure S6. SEM images of pristine (t_0) and degraded samples, after 2 (t_1), 5 (t_2), and 12 (t_3) days (scalebar 10 μm): a) SP samples; b) SP-1.0Fe samples.

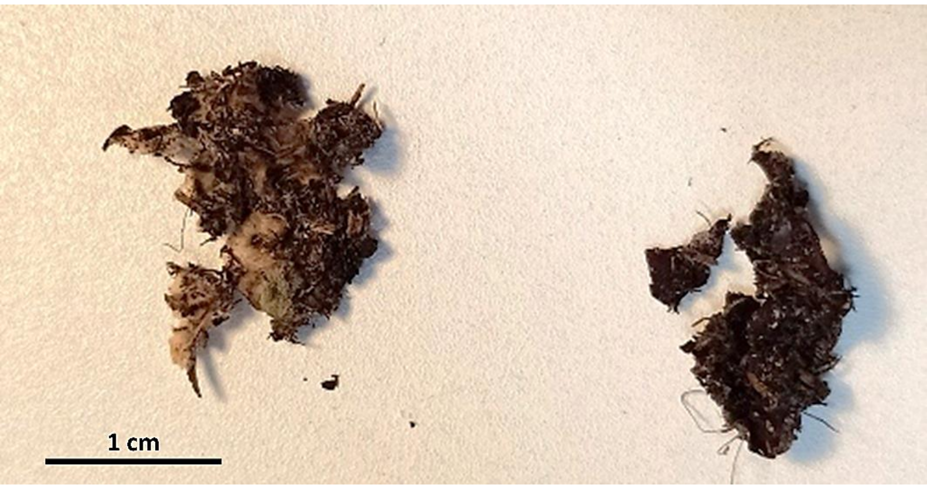

Figure S7. Image of a sample of SP (left) and SP-1.0Fe (right) retrieved at day 20 (t_4).

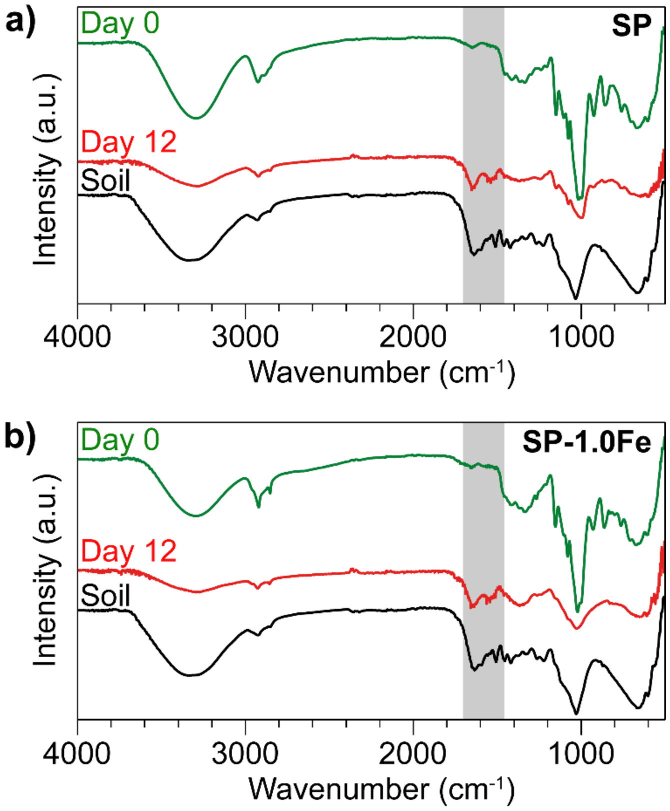

Figure S8. FTIR spectra of pristine (t_0), degraded sample after 12 (t_3) days, and soil. The grey shaded area corresponds to wavenumbers 1700-1450 cm^{-1}: a) SP samples; b) SP-1.0Fe samples.

Functional Properties of LIG

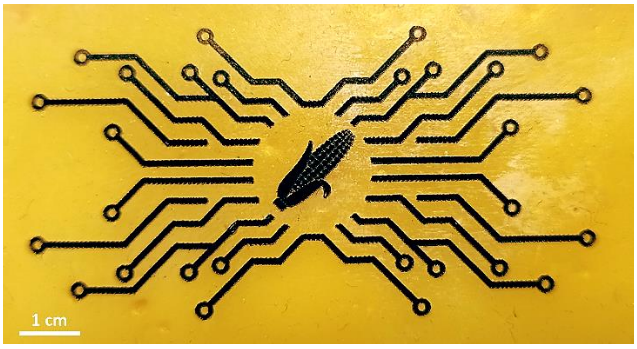

Figure S9. Proof of concept of a LIG circuit on SP-1.0Fe.

The correlation between resistance and relative humidity values is reported in **Table S7**.

The control sample (pristine SP-1.0Fe), showed a decrease in resistance for an increased humidity value, caused by the ionic conductivity of the swelled starch. The resistance values spanned from several MΩ to about 94 kΩ. At low humidity, starch can therefore be considered an insulating material.

The LIG sample, instead, showed the opposite behavior, with an increase in resistance for an increased humidity value. At high humidity, the sample is swelled by water and thus a mixed contribution to its electrical resistance is observed: from LIG (electronic) and starch (ionic). The resistance readings can be considered the equivalent resistance for the two resistors in parallel (LIG and starch). At low humidity, the starch contribution is negligible, and therefore the value obtained at 30% humidity can be considered a good estimation of the “true” LIG resistance.

Table S7. Resistance values of pristine and scribed SP-1.0Fe at T = 25 °C and different relative humidity levels for samples (resistors) with length 40 mm, and width 5 mm.

Relative humidity [%]	Resistance of pristine SP-1.0Fe [kΩ]	Resistance of scribed SP-1.0Fe [kΩ]
30	11579.7 ± 21922.0	59.4 ± 0.4
60	1264.3 ± 272.4	79.6 ± 0.9
90	94.3 ± 3.2	91.9 ± 2.6

Table S8. Comparison between the performances and applications of magnetic LIG in literature and this work.

Ref.	Precursor	Method	Magnetic Compounds	M_s [emu g^{-1}]	Application
[42]	polybenzoxazine + Fe(acac)$_3$	precursor mixing	Fe_3O_4	5.27	EM shielding
[43]	polyimide	Electrodeposition on LIG	Ni	30.3	EM shielding
[44]	polybenzoxazine	Fe(acac)$_3$ drop-casting on LIG + reirradiation	Fe_3O_4	14.4	EM shielding
	polybenzoxazine	Co(acac)$_3$ drop-casting on LIG + reirradiation	FeCo	36.3	EM shielding
This work	SP* + Fe(NO$_3$)$_3$	precursor mixing	γ-Fe_2O_3	10	Dye absorption

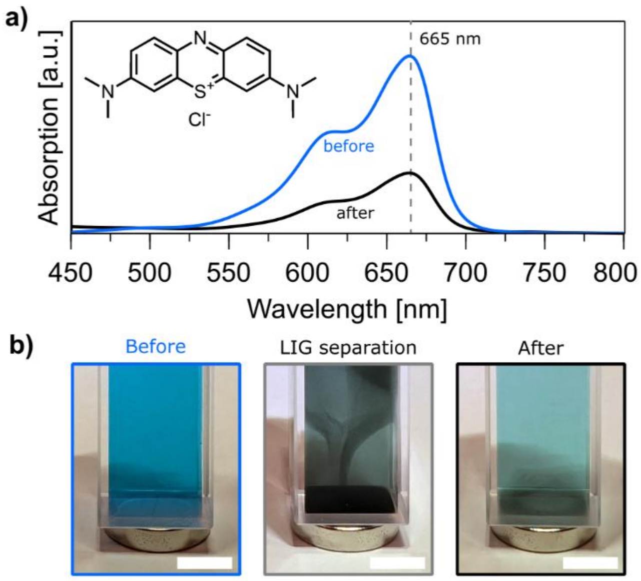

Figure S10. a) UV-Vis spectrum of the MB solution before and after the absorption via starch-based magnetic LIG, inset: chemical structure of methylene blue (MB); b) images of the MB solution before (left), during (center), and after (right) the absorption and magnetic separation of the magnetic LIG powder (scalebar 5 mm).

Bibliography

[1] M. Abdulhafez, G. N. Tomaraei, and M. Bedewy, “Fluence-Dependent Morphological Transitions in Laser-Induced Graphene Electrodes on Polyimide Substrates for Flexible Devices,” *ACS Appl. Nano Mater.*, vol. 4, no. 3, pp. 2973–2986, Mar. 2021, doi: 10.1021/acsanm.1c00101.

[2] C. H. Dreimol *et al.*, “Sustainable wood electronics by iron-catalyzed laser-induced graphitization for large-scale applications,” *Nat Commun*, vol. 13, no. 1, Art. no. 1, Jun. 2022, doi: 10.1038/s41467-022-31283-7.

[3] Y. Chyan, R. Ye, Y. Li, S. P. Singh, C. J. Arnusch, and J. M. Tour, “Laser-Induced Graphene by Multiple Lasing: Toward Electronics on Cloth, Paper, and Food,” *ACS Nano*, vol. 12, no. 3, pp. 2176–2183, Mar. 2018, doi: 10.1021/acsnano.7b08539.

[4] T. D. Le *et al.*, “Green Flexible Graphene–Inorganic-Hybrid Micro-Supercapacitors Made of Fallen Leaves Enabled by Ultrafast Laser Pulses,” *Adv Funct Materials*, vol. 32, no. 20, p. 2107768, May 2022, doi: 10.1002/adfm.202107768.

[5] R. Miyakoshi, S. Hayashi, and M. Terakawa, “Simultaneous laser-based graphitization and microstructuring of bamboo for supercapacitors derived from renewable resources,” *RSC Adv.*, vol. 12, no. 46, pp. 29647–29652, Oct. 2022, doi: 10.1039/D2RA05641K.

[6] S. L. Silvestre *et al.*, “Cork derived laser-induced graphene for sustainable green electronics,” *Flex. Print. Electron.*, vol. 7, no. 3, p. 035021, Sep. 2022, doi: 10.1088/2058-8585/ac8e7b.

[7] A. Imbrogno *et al.*, “Laser-Induced Graphene Supercapacitors by Direct Laser Writing of Cork Natural Substrates,” *ACS Appl. Electron. Mater.*, Mar. 2022, doi: 10.1021/acsaelm.1c01202.

[8] W. R. de Araujo, C. M. R. Frasson, W. A. Ameku, J. R. Silva, L. Angnes, and T. R. L. C. Paixão, “Single-Step Reagentless Laser Scribing Fabrication of Electrochemical Paper-Based Analytical Devices,” *Angewandte Chemie*, vol. 129, no. 47, pp. 15309–15313, Oct. 2017, doi: 10.1002/ange.201708527.

[9] B. Kulyk *et al.*, “Laser-Induced Graphene from Paper for Mechanical Sensing,” *ACS Appl. Mater. Interfaces*, vol. 13, no. 8, pp. 10210–10221, Mar. 2021, doi: 10.1021/acsami.0c20270.

[10] P. I. C. Claro *et al.*, “Tuning the Electrical Properties of Cellulose Nanocrystals through Laser-Induced Graphitization for UV Photodetectors,” *ACS Appl. Nano Mater.*, Jul. 2021, doi: 10.1021/acsanm.1c01453.

[11] B. Kulyk *et al.*, “Conversion of paper and xylan into laser-induced graphene for environmentally friendly sensors,” *Diamond and Related Materials*, vol. 123, p. 108855, Mar. 2022, doi: 10.1016/j.diamond.2022.108855.

[12] S. Lee and S. Jeon, “Laser-Induced Graphitization of Cellulose Nanofiber Substrates under Ambient Conditions,” *ACS Sustainable Chem. Eng.*, vol. 7, no. 2, pp. 2270–2275, Jan. 2019, doi: 10.1021/acssuschemeng.8b04955.

[13] W. Zhang, Y. Lei, F. Ming, Q. Jiang, P. M. F. J. Costa, and H. N. Alshareef, “Lignin Laser Lithography: A Direct-Write Method for Fabricating 3D Graphene Electrodes for Microsupercapacitors,” *Advanced Energy Materials*, vol. 8, no. 27, p. 1801840, 2018, doi: 10.1002/aenm.201801840.

[14] Y. Lin *et al.*, “Fabricating Nanodiamonds from Biomass by Direct Laser Writing under Ambient Conditions,” *ACS Sustainable Chem. Eng.*, vol. 9, no. 8, pp. 3112–3123, Mar. 2021, doi: 10.1021/acssuschemeng.0c07607.

[15] Y. Lei, A. H. Alshareef, W. Zhao, and S. Inal, “Laser-Scribed Graphene Electrodes Derived from Lignin for Biochemical Sensing,” *ACS Appl. Nano Mater.*, Dec. 2019, doi: 10.1021/acsanm.9b01795.

[16] J. Lin *et al.*, “Laser-induced porous graphene films from commercial polymers,” *Nature Communications*, vol. 5, Art. no. 1, Dec. 2014, doi: 10.1038/ncomms6714.

[17] L. Tang, J. Zhou, D. Zhang, and B. Sheng, “Laser-Induced Graphene Electrodes on Poly(ether–ether–ketone)/PDMS Composite Films for Flexible Strain and Humidity Sensors,” *ACS Appl. Nano Mater.*, vol. 6, no. 19, pp. 17802–17813, Oct. 2023, doi: 10.1021/acsanm.3c03026.

[18] G. M. da Costa, E. De Grave, and R. E. Vandenberghe, “Mössbauer studies of magnetite and Al-substituted maghemites,” *Hyperfine Interactions*, vol. 117, no. 1, pp. 207–243, Dec. 1998, doi: 10.1023/A:1012691209853.

[19] J. M. Orozco-Henao, D. Muraca, F. H. Sánchez, and P. M. Zélis, “Determination of the effective anisotropy of magnetite/maghemite nanoparticles from Mössbauer effect spectra,” *J. Phys. D: Appl. Phys.*, vol. 55, no. 33, p. 335302, Jun. 2022, doi: 10.1088/1361-6463/ac708e.

[20] B. David, N. Pizúrová, O. Schneeweiss, V. Kudrle, O. Jašek, and P. Synek, “Iron-Based Nanopowders Containing α-Fe, Fe3C, and γ-Fe Particles Synthesised in Microwave Torch Plasma and Investigated with Mössbauer Spectroscopy,” *Jpn. J. Appl. Phys.*, vol. 50, no. 8S1, p. 08JF11, Aug. 2011, doi: 10.1143/JJAP.50.08JF11.

[21] C. Değer, “Introduction to Magnetic Materials, 2nd edition”, Accessed: Jan. 15, 2024. [Online]. Available: https://www.academia.edu/20298510/Introduction_to_Magnetic_Materials_2nd_edition

[22] B. David, O. Schneeweiss, F. Dumitrache, C. Fleaca, R. Alexandrescu, and I. Morjan, “Powders with superparamagnetic Fe3C particles studied with Mössbauer spectrometry,” *J. Phys.: Conf. Ser.*, vol. 217, no. 1, p. 012097, Mar. 2010, doi: 10.1088/1742-6596/217/1/012097.

[23] X.-X. Bi, B. Ganguly, G. P. Huffman, F. E. Huggins, M. Endo, and P. C. Eklund, “Nanocrystalline α–Fe, Fe3C, and Fe7C3 produced by CO2 laser pyrolysis,” *Journal of Materials Research*, vol. 8, no. 7, pp. 1666–1674, Jul. 1993, doi: 10.1557/JMR.1993.1666.

[24] M. Kořenek, T. Ivanova, V. Svačinová, and M. Mašláň, “Mössbauer Study on the Conversion of Different Iron-Based Catalysts Used in Carbon Nanotube Synthesis,” *Nanomaterials*, vol. 13, no. 23, Art. no. 23, Jan. 2023, doi: 10.3390/nano13233010.

[25] X. Lei, W. Wang, Z. Ye, N. Zhao, and H. Yang, “High saturation magnetization of Fe3C nanoparticles synthesized by a simple route,” *Dyes and Pigments*, vol. 139, pp. 448–452, Apr. 2017, doi: 10.1016/j.dyepig.2016.12.046.

[26] Sarveena *et al.*, “Synthesis, phase composition, Mössbauer and magnetic characterization of iron oxide nanoparticles,” *Phys. Chem. Chem. Phys.*, vol. 18, no. 14, pp. 9561–9568, Mar. 2016, doi: 10.1039/C5CP07698F.

[27] L. Herojit singh, R. Govindaraj, R. Mythili, and G. Amarendra, “Stability and magnetic interactions between magnetite nanoparticles dispersed in zeolite as studied using Mössbauer spectroscopy,” *Journal of Magnetism and Magnetic Materials*, vol. 418, pp. 248–252, Nov. 2016, doi: 10.1016/j.jmmm.2016.04.024.

[28] H. Zhang, “The Mossbauer spectra of carbon-coated iron and iron compound nanocrystals produced by arc discharge,” *Journal of Materials Science Letters*, vol. 18, no. 11, pp. 919–920, Jun. 1999, doi: 10.1023/A:1006677017091.

[29] A. Mijovilovich, A. Gonçalves Vieira, R. Paniago, H. D. Pfannes, and B. Mendonça Gonzalez, “Mössbauer study of the retained austenitic phase in multiphase steels,” *Materials Science and Engineering: A*, vol. 283, no. 1, pp. 65–69, May 2000, doi: 10.1016/S0921-5093(00)00620-1.

[30] P. G. Rouxhet and M. J. Genet, “XPS analysis of bio-organic systems,” *Surface and Interface Analysis*, vol. 43, no. 12, pp. 1453–1470, 2011, doi: 10.1002/sia.3831.

[31] M. C. Biesinger, “Accessing the robustness of adventitious carbon for charge referencing (correction) purposes in XPS analysis: Insights from a multi-user facility data review,” *Applied Surface Science*, vol. 597, p. 153681, Sep. 2022, doi: 10.1016/j.apsusc.2022.153681.

[32] “High Resolution XPS of Organic Polymers: The Scienta ESCA300 Database (Beamson, G.; Briggs, D.),” *J. Chem. Educ.*, vol. 70, no. 1, p. A25, Jan. 1993, doi: 10.1021/ed070pA25.5.

[33] A. V. Naumkin, A. Kraut-Vass, C. J. Powell, and S. W. Gaarenstroom, *NIST X-ray photoelectron spectroscopy database*, Version 4.1. in NIST standard reference database. Gaithersburg, MD: Measurement Services Division of the National Institute of Standards and Technology (NIST) Technology Services, 2012. Accessed: Sep. 20, 2024. [Online]. Available: http://srdata.nist.gov/xps/Default.aspx

[34] M. C. Biesinger, B. P. Payne, A. P. Grosvenor, L. W. M. Lau, A. R. Gerson, and R. St. C. Smart, "Resolving surface chemical states in XPS analysis of first row transition metals, oxides and hydroxides: Cr, Mn, Fe, Co and Ni," *Applied Surface Science*, vol. 257, no. 7, pp. 2717–2730, Jan. 2011, doi: 10.1016/j.apsusc.2010.10.051.

[35] B. V. Crist, "Handbooks of Monochromatic XPS Spectra".

[36] A. P. Grosvenor, B. A. Kobe, M. C. Biesinger, and N. S. McIntyre, "Investigation of multiplet splitting of Fe 2p XPS spectra and bonding in iron compounds," *Surface and Interface Analysis*, vol. 36, no. 12, pp. 1564–1574, 2004, doi: 10.1002/sia.1984.

[37] I. Uhlig, R. Szargan, H. W. Nesbitt, and K. Laajalehto, "Surface states and reactivity of pyrite and marcasite," *Applied Surface Science*, vol. 179, no. 1, pp. 222–229, Jul. 2001, doi: 10.1016/S0169-4332(01)00283-5.

[38] W. Yu, Y. Peng, L. Cao, W. Zhao, and X. Liu, "Free-standing laser-induced graphene films for high-performance electromagnetic interference shielding," *Carbon*, vol. 183, pp. 600–611, Oct. 2021, doi: 10.1016/j.carbon.2021.07.055.

[39] J. Yin *et al.*, "Flexible 3D porous graphene film decorated with nickel nanoparticles for absorption-dominated electromagnetic interference shielding," *Chemical Engineering Journal*, vol. 421, p. 129763, Oct. 2021, doi: 10.1016/j.cej.2021.129763.

[40] Z. Yu, W. Yu, Y. Jiang, Z. Wang, W. Zhao, and X. Liu, "Upcycling of Polybenzoxazine to Magnetic Metal Nanoparticle-Doped Laser-Induced Graphene for Electromagnetic Interference Shielding," *ACS Appl. Nano Mater.*, vol. 5, no. 9, pp. 13158–13170, Sep. 2022, doi: 10.1021/acsanm.2c02912.

[41] Y. Jiang *et al.*, "Reusable, magnetic laser-induced graphene for efficient removal of organic pollutants from water," *Carbon Lett.*, Mar. 2022, doi: 10.1007/s42823-022-00336-8.

[42] W. Yu, Y. Peng, L. Cao, W. Zhao, and X. Liu, "Free-standing laser-induced graphene films for high-performance electromagnetic interference shielding," *Carbon*, vol. 183, pp. 600–611, Oct. 2021, doi: 10.1016/j.carbon.2021.07.055.

[43] J. Yin *et al.*, “Flexible 3D porous graphene film decorated with nickel nanoparticles for absorption-dominated electromagnetic interference shielding,” *Chemical Engineering Journal*, vol. 421, p. 129763, Oct. 2021, doi: 10.1016/j.cej.2021.129763.

[44] Z. Yu, W. Yu, Y. Jiang, Z. Wang, W. Zhao, and X. Liu, “Upcycling of Polybenzoxazine to Magnetic Metal Nanoparticle-Doped Laser-Induced Graphene for Electromagnetic Interference Shielding,” *ACS Appl. Nano Mater.*, vol. 5, no. 9, pp. 13158–13170, Sep. 2022, doi: 10.1021/acsanm.2c02912.
